# Internet of Food (IoF), Tailor-Made Metal Oxide Gas Sensors to Support Tea Supply Chain

**DOI:** 10.3390/s21134266

**Published:** 2021-06-22

**Authors:** Estefanía Núñez-Carmona, Marco Abbatangelo, Veronica Sberveglieri

**Affiliations:** 1 CNR-IBBR, Institute of Bioscience and Bioresources, Via Madonna del Piano, 10, 50019 Sesto Fiorentino, FI, Italy; estefania.nunezcarmona@ibbr.cnr.it (E.N.-C.); veronica.sberveglieri@ibbr.cnr.it (V.S.); 2 Nano Sensor Systems, NASYS Spin-Off University of Brescia, Brescia, Via Camillo Brozzoni, 9, 25125 Brescia, BS, Italy

**Keywords:** volatiloma, tea, MOX sensors, S3, GC-MS

## Abstract

Tea is the second most consumed beverage, and its aroma, determined by volatile compounds (VOCs) present in leaves or developed during the processing stages, has a great influence on the final quality. The goal of this study is to determine the volatilome of different types of tea to provide a competitive tool in terms of time and costs to recognize and enhance the quality of the product in the food chain. Analyzed samples are representative of the three major types of tea: black, green, and white. VOCs were studied in parallel with different technologies and methods: gas chromatography coupled with mass spectrometer and solid phase microextraction (SPME-GC-MS) and a device called small sensor system, (S3). S3 is made up of tailor-made metal oxide gas sensors, whose operating principle is based on the variation of sensor resistance based on volatiloma exposure. The data obtained were processed through multivariate statistics, showing the full file of the pre-established aim. From the results obtained, it is understood how supportive an innovative technology can be, remotely controllable supported by machine learning (IoF), aimed in the future at increasing food safety along the entire production chain, as an early warning system for possible microbiological or chemical contamination.

## 1. Introduction

Tea is native to the northern hills at the foot of the Himalayas where the inhabitants chewed *Camellia sinensis* for medicinal purposes. The Chinese populations then invented preservation techniques to increase the shelf life and facilitate the transport of the product. Over time, the techniques have improved, and the cultivation areas have increased so as to arrive at the point today where many varieties are known [1].

It is one of the most consumed beverages worldwide, with an increase in the consumption of 23.4% in the last 7 years, reaching 297 billion liters by 2021. Generally, tea is an aromatic beverage prepared by pouring hot or steaming water over dried or fresh leaves of the *Camellia sinensis* (green, white, and Oolong teas), an evergreen shrub (bush) and *Camellia assamica* (black and Pu-Erh tea), which originate and are cultivated, respectively, in China and India [2,3].

The most produced and consumed teas worldwide are green and black teas [4].

Tea is an infusion, but not all infusions are tea, as there are infusions or herbal teas that originate from red fruits, chamomile, mint, lavender, etc.

The tea leaves undergo different treatments that determine their classification. Spices or herbs are added to the basic tea in order to spice up the flavor and taste. The first classification is based on the fermentation treatment and is also the classification used at customs; in particular, we have:

- Fermented tea: black and Pu-Erh tea
- Unfermented tea: green and white tea,
- Partially or semi-fermented tea: Oolong tea.

In addition to this classification, the teas are placed in other classes based on the following specific treatments: scented tea, flavored tea, smoked tea, blends, pressed teas, and a bouquet of tea and flowers.

There are many distinctive types of tea; some, such as Darjeeling, Ceylon, Oolong, etc., have cooling, slightly bitter, and astringent flavors, [5,6,7,8] while others have vastly different profiles that include sweet, nutty, floral, or grassy notes.

Tea leaves contain thousands of chemical compounds and release volatile compounds (VOCs), which contribute to the definition of product quality [9,10,11,12,13,14].

The aroma is one of the determining factors in the quality of tea and is due to the volatile compounds. Volatile compounds are mainly responsible for the flavor and aroma of the infusion, and many of them are not present in the fresh leaf but develop after processing.

There are more than 600 volatile compounds (VCs) in tea, resulting from the enzymatic action on odorless compounds present in the leaf that are released after rolling and fermentation [15]. The volatile aromatic compounds differ according to the types of tea, both for the “different” fresh material and for different production processes, without forgetting that the final consumer requires that the purchased product be recognized and standardized.

Recent research has shown that the volatile aromatic components of tea are influenced by several factors: cultivar, area of cultivation, cultural practices, production methods, and conservation [16,17,18,19].

Many kinds of classical analytical chemical techniques such as the gas chromatography coupled with mass spectrometry (GC-MS) have been largely used and have demonstrated their accuracy and specificity, but present important limits are normally expensive and time consuming and require appropriately trained staff to operate them.

On the other hand, an innovative tailor-made gas sensor device named small sensor systems (S3) has been applied broadly in the quality control in food the field and environmental monitoring as well exhibiting remarkable results [20,21,22].

This innovative device, S3, is fast, remotely controllable, totally user friendly, and, once trained, does not need any special skilled staff to operate it. In particular the S3 device is totally tailor-made for the specific application. That is, the sensors are grown, calibrated, and used on the basis of the class of VOCs on which they will be used, thus managing to obtain greater sensitivity and accuracy.

It can then be easily inserted into the production chain, for the evaluation of quality standards or to follow, for example, the evolution of the product over time.

The goal of this study is the characterization of the aroma of different types of tea and the definition of their volatiloma through the use of two different approaches, to support the supply chain of tea. Providing a portable device capable of monitoring a greater quantity of product at low costs and very quickly in a noninvasive way to facilitate compliance with quality standards.

## 2. Materials and Methods

The samples taken into consideration belong to 3 more consumed worldwide types of tea: black tea, green tea, and white tea. In this study, 20 mL chromatographic vials were used, each filled with approximately one sachet, 2 ± 0.2 g of tea. The used sample, the code name, and the number of replicas used for each technique are represented in Table 1.

During the sampling process, no chemical extraction or thermal shock was carried out on the samples in order to keep the aroma of the dried product, to evaluate its actual characteristics. The vials were closed with aluminum caps containing polytetrafluoroethylene (PTFE) and silicone septa. The operational conditions were interpreted in Section 2.2, respectively.

### 2.1. GC-MS Analysis Conditions

After closure, vials were placed in the autosampler HT280T (HTA s.r.l., Brescia, Italy) to proceed with vial conditioning and volatile organic compound (VOC) extraction.

Conditioning of the sample was performed as follows: filled vials were maintained for 15 min at 40 °C in order to equilibrate the headspace (HS) of the sample and to remove any variables. Afterward, VOCs extraction was performed using solid-phase microextraction (SPME) analysis, and the fiber used for the adsorption of volatiles was a divinylbenzene/carboxen/polydimethylsiloxane (DVB/CAR/PDMS) 50/30 µm (Supelco Co. Bellefonte, PA, USA) placed on the HT280T autosampler. The fiber was exposed to the vial HS in the HT280T oven thermostatically regulated at 40 °C for 15 min.

The GC instrument used in this work was a Shimadzu GC 2010 PLUS (Kyoto, KYT, Japan), equipped with a Shimadzu single quadrupole mass spectrometer (MS) MS-QP2010 Ultra (Kyoto, KYT, Japan). Fiber desorption took place in the GC–MS injector for 6 min at 250 °C. GC was operated in the direct mode throughout the run, while the separation was performed on a MEGA-WAX capillary column, 30 m × 0.25 mm × 0.25 μm film thickness, (Agilent Technologies, Santa Clara, CA, USA). Hydrogen was used as the carrier gas and has been produced by GENius PF500, FullTech Instruments Srl. (Rome, Italy) at a constant flow rate of 2.34 mL/min.

The GC oven temperature programming was applied as follows: at the beginning, the chromatographic column was held at 40 °C for 2 min and, subsequently, the temperature was raised from 40 to 100 °C at 2 °C/min. Next, the temperature was raised from 100 to 180 °C, with a rate of 5 °C/min; finally, the temperature was raised from 180 to 230 °C at a rate of 10 °C/min and was maintained for 5 min, for a total program time of 58 min [21,23,24].

During the analysis, the GC–MS interface was kept at 200 °C, with the mass spectrometer in the electron ionization (EI) mode (70 eV) and related to instrument tuning, and the ion source was kept at 200 °C. Mass spectra were collected over 35 to 500 *m*/*z*, in a range in the total ion current (TIC) mode, with scan intervals at 0.3 s. VOC identification was carried out using the NIST11 and the FFNSC2 libraries of mass spectra.

Chromatogram peak integration was performed using the peak area as target parameter programming an automatic integration round, using 70 as the minimum number of peak detection and 500 as the minimum area to detect. Other parameters used in the automatic peak integration were slope 100/min, width 2 s, drift 0/min, and doubling time (T.DBL) 1000 min, and no smoothing method was applied. The final round of peak integration was performed by manual peak integration for all the obtained chromatograms.

### 2.2. S3 Analysis Conditions

The chosen sensors for the application were installed on the S3 device, an acronym that stands for small sensor system.

The device that was designed and built by Nasys S.r.l. (www.nasys.it, accessed on 21 June 2021) is an innovative spin-off born in the University of Brescia, where tailor-made sensors were produced.

S3 is composed of three essential parts: (A) sensors chamber, (B) fluid dynamic circuit for the distribution of volatile compounds, and (C) electronics control system.

(A) The sensors are housed inside a steel chamber isolated from the external environment, except for an inlet and an outlet path for the passage of volatile compounds. In addition to the MOX sensors, a temperature, humidity sensor, and a flow sensor are also allocated as necessary to take into account the number of variables during the analysis. The dimensions of the chamber are 11 × 6.5 × 1.3 cm.

On the 11 and 6.5 cm sides, 6 and 5 positions have been obtained, respectively: 10 positions were available for the use of metal oxide (MOX) sensors and one for the temperature and humidity sensor. The list of MOX used in this study is represented in Table 2, where the technology of production, RGTO (rheotaxial growth and thermal oxidation) or nanowire, the sensing material (SnO_2_ or CuO), and the working temperature are indicated.

(B) The fluid dynamic circuit consists of a pump (Knf, model: NMP05B), polyurethane pipes, a solenoid valve, and a metal cylinder containing activated carbon for filtering possible interfering odors present in the environment. The pump flow is regulated by a needle valve placed at the chamber inlet; the flow range for tea analysis was set to 100 sccm.
(C) The electronic boards make it possible to acquire the resistances of the sensors, the correct heating of the sensors themselves to their operating temperature, and the sending of data to the Web App dedicated to the S3 device through an internet connection. In addition, it allows communication and synchronization with an autosampler. This is an autosampler of the company HTA S.r.l. (model HT2010H) which allows one to prepare batches of 42 samples per measurement session. Tea samples were conditioned for 5 min at 40 °C with 1 min in a shaking mode in order to equilibrate the headspace (HS).

The RGTO technique requires two phases of deposition: the first step is the metallic thin film by DC magnetron sputtering from a metallic target to a substrate at higher temperatures than the melting point of the metal; and the second step is the thermal oxidation period in order to produce a metal oxide coating with stable stoichiometry [25]. The surface of the thin film is rough, and this is desirable since it has a high surface-to-volume ratio and reactivity to the gaseous species [26]. In addition, the presence of this very rough surface morphology, also known as ‘spongy agglomerates’, gives rise to a highly specific area required for high-sensitivity gas sensors [27]. Nanowires display extraordinary crystalline quality and a very high length-to-width ratio, resulting in improved sensitivity and long-term material stability for extended operation [28,29]. The fabrication method consists of the evaporation of the powder (metal oxide) at high temperatures in a controlled atmosphere at pressures of less than a hundred mbar (50–200 mbar) and the subsequent mass transfer of the vapor (50–100 sccm) to substrates held at lower temperatures in relation to the evaporation source area. This growth technique is called a vapor–liquid–solid (VLS) mechanism.

For SnO_2_ sensors, the powders are mounted in the middle of the furnace at 1370 °C, and the inert air flow at temperatures between 350 and 400 °C is used as a carrier from the furnace to the substrate where nanowires begin to develop [30].

S3 was previously used, with considerable success, in numerous studies applied to the field of food technology and quality control [31,32]. The output of the S3 analysis consists of the sensors’ resistance variation due to the interaction of VOCs with the sensing elements. The exposure to VOCs lasted 1 min, while 9 min passed to restore sensors’ baseline. Prior to analysis, the sensors’ responses in terms of resistance (Ω) were standardized relative to the first value of the acquisition (R0). This standardization was performed for each measure so that for all the sensors, the first point value was 1, smoothing differences in the starting value of resistances between the measures themselves. For all the sensors, the difference between the first value and the minimum value was determined during the time of analysis; thus, the value ΔR/R0 was derived. These features were used as input for principal component analysis (PCA). Here, PCA has been used to visualize the data cluster. By contrast, a hard approach to quantify the accuracy of the system was employed. In particular, the k-nearest neighbor (k-NN) algorithm was used with 5-fold crossvalidation technique. The aim of crossvalidation is to test the model’s ability to predict new data that were not used in estimating it, in order to counteract overfitting or selection bias. The accuracy provided is the mean of the accuracies of the 5 steps of prediction.

## 3. Results

### 3.1. GC-MS Results

After closure, vials were placed in the autosampler HT280T (HTA s.r.l., Brescia, Italy) to proceed with vial conditioning and volatile organic compound (VOC) extraction.

Regarding the data extracted from the GC-MS-SPME analysis, the volatile fingerprints for each tea were identified, and the tables are presented at the end of the article in Appendix A (Table A1, Table A2, Table A3, Table A4, Table A5, Table A6, Table A7, Table A8, Table A9, Table A10 and Table A11). It was possible to identify the common compounds between the green tea samples that are presented in Table 3.

In total, an average of 100 volatile compounds were found for each sample analyzed, of which only seven in common to all samples.

- Cyanoacetic acid: (C_3_H_3_NO_2_) is an organic compound that has two functional groups: COOH typical of carboxylic acids and NC with triple bond typical of nitriles. It is obtained from the treatment of chloroacetate with sodium cyanide followed by acidification or electrolysis by cathodic reduction of carbon dioxide or the anodic oxidation of acetonitrile. It is a precursor of synthetic caffeine by theophylline [33].
- Hexanal: (C_6_H_10_O) is an aldehyde. In the cell, it is contained in the cytoplasm. It has a sweet almond and honey flavor and is found in several foods including soy, cucumber, black elderberry, and black currant [33].
- Limonene: (C_10_H_16_) is the most widespread and most important monoterpene. It has a lemon smell and turpentine-like notes. It is obtained by steam distillation of citrus peel and pulp obtained from the production of juice [33]. It should be specified that it is present as two isomers: R-LIMONENE and D-LIMONENE. In particular, it is R-LIMONENE in the SGP, while in the others, it is D-LIMONENE, and both isomers are present in the SGM.
- 6-methyl, 5-hepten-2-one: (C_8_H_14_O) is an unsaturated ketone called sulcatone. It has a strong, greasy, green, citrus smell and tastes reminiscent of pear. It is obtained from citronella or citral oil by mixing for 12 h in aqueous solution with K2CO3 and subsequent distillation and fractionation under vacuum. It was originally identified in lemongrass; later, it was also discovered in the essential oils of lemons and geraniums. We also find this ketone in grapes, melon, peaches, avocados, cognac, mangoes, rice, olives, blueberries, and more [33].
- Nonanal: (C_9_H_18_O) is an aldehyde. It has a strong and greasy odor which develops notes of orange and rose when diluted. The fat recalls the flavor of citrus fruits. It is synthesized by the catalytic oxidation of the corresponding alcohol or by the reduction of the respective acid. In nature, we find it in orange, mandarin, lemon, and lime oils. It is also found in more than 200 foods and beverages including apples, tomatoes, rum, wine, plum, coconut, cardamom, avocado, corn oil, broccoli, milk, eggs, tea, and others [34].
- α-Terpineol: (C_10_H_18_O) is a monoterpenic alcohol. It has a characteristic smell of lilac with a sweet flavor reminiscent of peach. It is obtained from the hydration of the terpene or from the pentane tricarboxylic acid by cyclization or from the isoprene and methyl-vinyl-ketone. It is present in more than 150 derivatives of herbs, leaves, and flowers. Form D is found in cardamom, star anise, sage, and marjoram oil. The L form is present in lavender, lime, and cinnamon leaves. The racemic form is the eucalyptus [33,34].
- 5,6,7,7a-Tetrahydro, 4,4,7a-Trimethyl-2(4H)–benzofuranone: (C_11_H_16_O_2_) is a heterocyclic compound. It has a coumarin and musky smell. This compound is formed from the photo-oxidation of carotene. The flavor is linked to the fruit and in particular to their point of ripeness. It is obtained from the degradation process of β-carotene in the presence of nitrogen and air. It occurs naturally in lemongrass and sweet grass oil [33].

On the other hand and regarding the GC-MS results from the black tea samples, it is possible to say that there were seven black teas subjected to GC-MS analysis, with different flavors and belonging to different origins and composition. The results obtained showed that there are four VOCs in common to all the samples (Table 4).

- Nonanal: (C_9_H_18_O) is an aldehyde. It has a strong and greasy odor which develops notes of orange and rose when diluted. The fat recalls the flavor of citrus fruits. It is synthesized by the catalytic oxidation of the corresponding alcohol or by the reduction of the respective acid. In nature, we find it in orange, mandarin, lemon, and lime oils. It is also found in more than 200 foods and drinks including apples, tomatoes, rum, wine, plum, coconut, cardamom, avocado, corn oil, broccoli, milk, egg, tea, and others [34].
- Ammonium acetate: (C_2_H_7_NO_2_) is an ammonium salt obtained from the reaction between ammonia and acetic acid. It is used to regulate acidity in food, even though the EU decided to ban its use as a food additive [33].
- Phenylethyl alcohol: (C_8_H_10_O) is an alcohol. It has a characteristic rose odor and initially a slight bitter taste. The dessert is reminiscent of peaches. It is synthesized from toluene, benzene, or styrene. It is found in esterified form in rose concentrate or distilled rose water. It is present in the essential oil of lily, narcissus, and tea leaves but not only because it has been found in more than 200 foods and drinks including peaches, grapes, coffee, tea, mushrooms, mango, kiwi, rum, whiskey, milk, butter, cheese, and more [33,34].
- 5,6,7,7a-Tetrahydro, 4,4,7a- trimethyl 2 (4H)—benzofuranone: (C_11_H_16_O_2_) is a heterocyclic compound. It has a coumarin and musky smell. This compound is formed from the photo-oxidation of carotene. The flavor is linked to the fruit and in particular to their point of ripeness. It is obtained from the degradation process of β-carotene in the presence of nitrogen and air. It occurs naturally in lemongrass and sweet grass oil [33].

### 3.2. S3 Results

**Figure 1:**
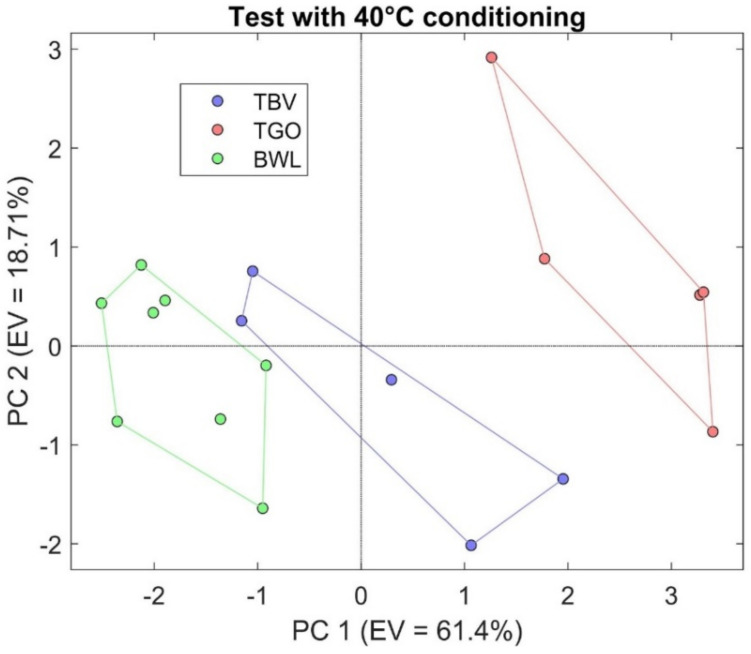
PCA analysis representing the results obtained from the comparison between black, green, and white tea.

For each analyzed tea type (black and green), a specific matrix was created as shown in the respective PCA scores plot. Conversely, the dataset used in the first PCA was obtained by joining the three tea types considered in an initial test to check the performances of the system. In Figure 1, the results obtained from the comparative analysis of the S3 for the samples belonging to the three types of teas BWL (white), TGO (green), and TBV (black) are represented.

**Figure 2:**
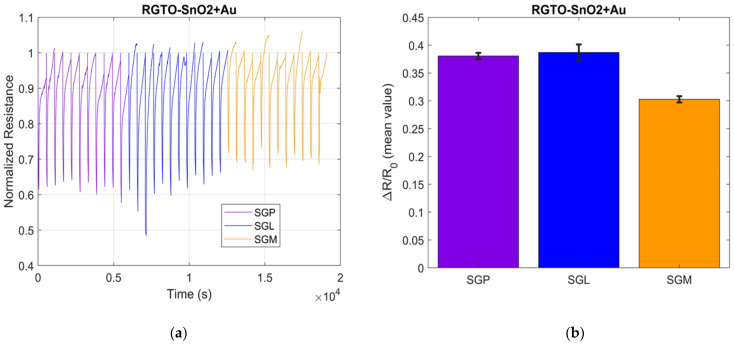
(**a**) Sensor response representation to the green teas RGTO sensor SnO_2_ + Au and (**b**) mean values for the ΔR/R0 with the representation of the SD bar for the different green teas measurements.

Total explained variance reached a value of 80.11%, exhibiting a good cluster separation between the three different categories of teas. On the other hand, further exploration of the data was conducted proceeding the comparison of different green and black teas in order to explore the capacity of the instrument to distinguish the same type of tea (black or green) but with different treatments and/or aromatization. In Figure 2, the results of the analysis of SGP, SGL, and SMG for one RGTO sensor as the normalized resistance as a function of time (left) are represented; these results are more evident in the bar chart where the line on the bars is the indicated standard deviation of the mean (Figure 2b).

**Figure 3:**
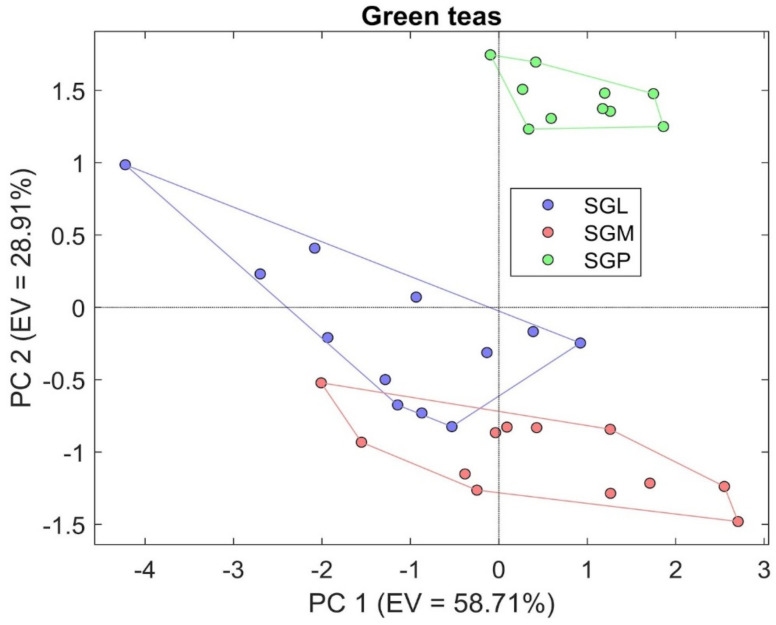
PCA analysis representing the results obtained from the comparison between green tea.

Regarding the result obtained, applying multivariate analysis to the signals obtained from all the sensors when measuring the types of green tea is represented in Figure 3.

The result obtained is very satisfactory, because considering the two main components, the total explained variance enclosed in the graph reaches 87.82%. The mean accuracy achieved with k-NN (k = 5) was equal to 88.57%.

**Figure 4:**
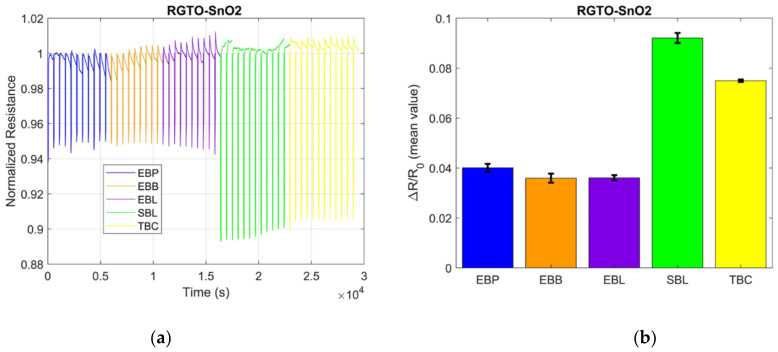
(**a**) Sensor response representation to the green teas RGTO sensor SnO_2_ and (**b**) mean values for the ΔR/R0 with the representation of the SD bar for the different green teas measurements.

Regarding the black teas, the samples that were taken into consideration were EBP, EBL, EBB, SBL, and TBC. Figure 4a,b clearly shows the different responses of a SnO_2_ RGTO sensor to the various black tea samples.

**Figure 5:**
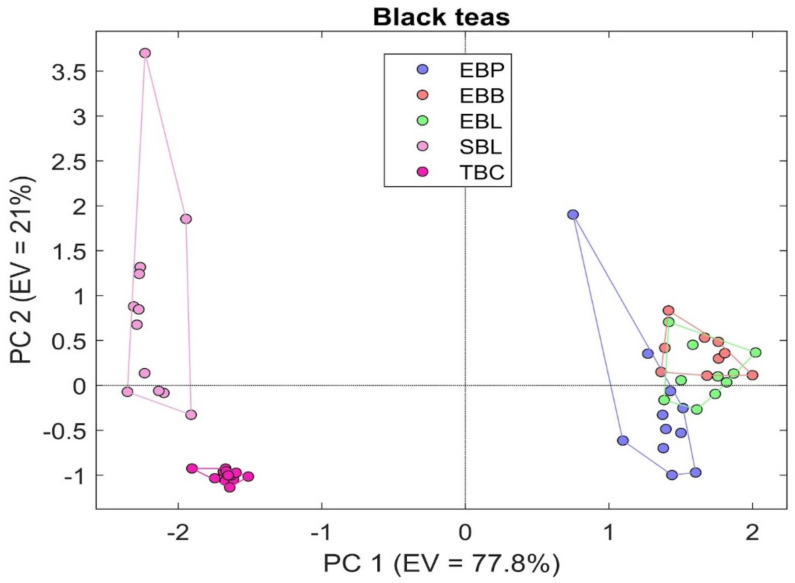
PCA analysis representing the results obtained from the comparison between black tea.

As before, PCA was then applied, and the results can be seen in Figure 5. The expected results were confirmed using PCA analysis, obtaining a good cluster separation, apart from the EBL, EBB, and EBP. The mean accuracy achieved with k-NN (k = 5) was equal to 83.45%.

## 4. Discussion

Regarding the GC-MS results from the treated green teas, an average of 100 volatile compounds were found for each sample analyzed, of which only seven in common to all samples (Table 3). As can be seen from the description, all are naturally occurring compounds in various foods and drinks, especially fruit and vegetables, except for cyanoacetic acid which is a precursor of synthetic caffeine. Volatile compounds give the tea floral, fruity, vegetable, spicy, and aromatic notes. On the other hand, regarding the GC-MS results from the black tea samples, seven teas were subjected to GC-MS analysis, with different characteristics. The results obtained showed that there were four VOCs in common to all the samples (Table 4). In general, EBP, SBL, and TBC were present in more than 100 VOCs, but in the others, about 90 have been found. The few compounds common to black teas can be explained by vanilla flavored black tea (TBV), which has fewer compounds in common with the other samples.

The four citrus-flavored samples (TBL, EBB, EBL, and SBL) have five compounds in common over the previous four: β-myrcene, D-limonene, Benzaldehyde, α-terpineol, and Carvone, which are present in citrus fruits both in essential oils and in peels. In TBV, there is a maximum peak in the direction of vanillin, which is the aromatic aldehyde that gives the vanilla aroma, which records an abundance of 8.287 × 10^6^. In general, even in black teas, as already found in green teas, volatile compounds are present in nature and impart aromas that have floral, vegetable, spicy, fruity, and aromatic notes.

Nonetheless, the results obtained throughout the use of the sensor device show a good rate of identification for the teas, since more than the 80% of the explained variance was enclosed between PC1 and PC2, and good clustering capacity can be seen in Figure 2.

Taking into consideration the response of one of the RGTO sensors for the green teas’ analysis, in particular SGP, SGL, and SMG, it is evident, in the graphs of the normalized resistance as a function of time (Figure 2a), how the samples are well separated according to the different aromatization, even if some have similar values. In fact, as can be seen in Figure 2a, the violet and blue values are more similar to each other. It looks even better in bar charts where the line on the bars is the indicated standard deviation of the mean, highlighting its reproducibility (Figure 2b).

On Figure 3, the results of the PCA analysis performed on green teas measurements is shown, and it is evident the separation between the green tea samples, in particular SGP, is far from SGL and SGM. This result is important not only because the instrument is able to recognize the aromatization tea from the pure one, separating the 2 different ones which actually slightly overlap. The result obtained is very satisfactory, because considering the two main components, the total explained variance enclosed in the graph reaches 87.82%.

On the other hand, EBP, EBL, EBB, SBL, and TBC samples were taken into consideration for the analysis of the sensors’ response. Figure 4a,b clearly shows the different responses of a SnO2 RGTO sensor to the various black tea samples. This aspect is even more evident in the bar graphs (Figure 4b). It can be observed that the samples of EBP, EBB, and EBL, all of the same brand, have a variation that is very similar to each other, while the other two (SBL and TBC) have a very different variation from the first three and are also quite different between themselves. In this case, two samples of black tea aromatized with lemon were taken into consideration but of different brands, and as can be seen in the figures, the two are very different from each other, as the variation associated with SLB tea is much greater than that of ELB tea.

PCA analysis multivariate analysis for black teas (Figure 5) confirms the previous discussed data, obtaining a good cluster separation, apart from the EBL, EBB, and EBP. These last three samples belong to the same brand and have been reported previously from the sensor response to have a lower sensor response than the other two samples of black tea and consequently are all close and partly overlapping each other, in particular EBB and EBL. This last consideration can be explained by observing the chromatograms where it can be seen how EBB and EBL are mainly characterized by limonene and linalyl acetate, while in EBP, the spectrum has a net peak in correspondence with linalyl acetate and has in general a chromatogram that appears to be richer than the previous two. In this case, the explained variability enclosed by the main components greater than the one obtained for the green teas, in fact, is 98.8%, and also in this case, the greatest variation explained is always along the PC1.

## 5. Conclusions

The work was based on the analysis of 11 different samples of green, black, and white tea. By applying the two techniques GC-MS and S3, the complete determination of the aromatic profile of the teas was achieved, evaluating and highlighting the similarities and differences between them so as to arrive at a discrimination based on the VOCs profile. Nearly, 100 different volatiles were identified by the mean in each tea sample. The GC-MS requires a longer and, in some ways, more elaborate analysis, while S3 allows one to obtain the result in a shorter time and more easily and user-friendly way. It can be concluded that an innovative technology such as S3 has the potential to be used from farm to fork, in food companies and in the production chain, so as to report anomalous products and prevent them from reaching the market or in any case arriving at a final product that must be excluded because it is not safe. In fact, once the anomaly is reported, it would be possible to implement a correction or exclusion of the batch from the distribution chain. It should also be emphasized that the use of this sensor technology once trained would have a positive economic impact for the production business, is also easy to use, and, thanks to the connection to the network, allows for remote data processing. In a short-term future, this technology could be applied in industrial reality, so that food safety and the producer can benefit from it. This kind of technology could be also implemented in household environments, to control not only the dried product but also the final stage of the beverage. In fact, its use is not limited only to tea but can concern any foodstuff, as already demonstrated in other studies.

## Figures and Tables

**Table 1:** Samples description.

Kind	Code	Description	Components	Number of Replicates	
				GC-MS	S3
Green tea	TGO	Green tea with orange aroma	88% Green tea + 10% natural orange aroma + 1% loto flower + 1% orange skin	3	5
	SGP	Pure green tea	100% Green tea	3	12
	SGL	Green tea with lemon aroma	Green tea 83% + lemon aroma + lemon juice concentrate 4%	3	12
	SGM	Green tea with Matcha tea	Green tea + green tea Matcha	3	12
Black tea	TBV	Black tea with vanilla aroma	91,5% tea + 8% aroma + 0,5% vanilla	3	5
	TBC	Pure black tea from Ceylon	100% Black tea Ceylon Sri Lanka	3	12
	EBL	Black tea with lemon aroma	Black tea from India and lemon aroma	3	12
	EBB	Black tea Earl Grey	Black Tea from India + bergamot juice	3	12
	EBP	Pure black tea	Biological black Tea	3	12
	SBL	Black tea with lemon aroma	Black tea + aroma + 1.04% powder of lemon juice	3	12
White tea	BWL	White tea with lemongrass	White tea leaf 90%, dried lemongrass 10%	3	8
Total number of samples for each technique				33	114

**Table 2:** Sensor array composition of S3 device and sensors characteristics.

Material	Kind	Working Temperature
SnO_2_ + Au	RGTO	400 °C
SnO_2_	RGTO	400 °C
CuO	Nanowire	350 °C
SnO_2_ + Au	Nanowire	350 °C
SnO_2_	Nanowire	350 °C

**Table 3:** Common compounds between the analyzed green teas.

Compound	TGO	SPG	SGL	SGM
Cyanoacetic acid	1.685 × 10^5^	1.165 × 10^5^	5.677 × 10^4^	6.301 × 10^4^
Hexanal	7.22 × 10^5^	5.751 × 10^5^	2.889 × 10^4^	3.529 × 10^5^
Limonene	1.378 × 10^7^	1.976 × 10^5^	2.602 × 10^8^	2.011 × 10^5^
6-metile 5-epten-2-one	6.233 × 10^5^	3.789 × 10^5^	2.731 × 10^5^	3.617 × 10^5^
Nonanal	5.81 × 10^5^	1.133 × 10^5^	9.298 × 10^4^	1.462 × 10^5^
α-terpineol	1.098 × 10^6^	3.145 × 10^6^	8.024 × 10^6^	7.628 × 10^5^
5,6,7,7a-tetrahydro, 4,4,7a- Trimethyl 2 (4H)—benzofuranone	3.448 × 10^5^	4.843 × 10^5^	4.819 × 10^5^	2.836 × 10^5^

**Table 4:** Common compounds between the analyzed black teas.

Compound	TBV	EBP	EBL	EBB	SBL	TBC
Nonanal	3.739 × 10^5^	6.164 × 10^4^	2.278 × 10^5^	1.129 × 10^5^	1.041 × 10^5^	1.198 × 10^5^
Ammonium acetate	2.141× 10^6^	4.641 × 10^4^	5.945 × 10^5^	6.868 × 10^5^	2.162 × 10^5^	1.023 × 10^5^
Phenylethyl alcohol	8.167× 10^4^	4.682 × 10^4^	4.243 × 10^4^	7.557 × 10^4^	6.461 × 10^4^	5.550 × 10^4^
5,6,7,7a-tetrahydro, 4,4,7a- Trimethyl 2 (4H)—benzofuranone	2.784 × 10^5^	3.536 × 10^4^	1.473 × 10^5^	7.543 × 10^4^	1.073 × 10^5^	1.053 × 10^5^

## Data Availability

Data available on request due to restrictions e.g., privacy or ethical.

## Appendix A

In Appendix A, the data are presented that were extracted from the GC-MS-SPME analysis of the volatile fingerprints for each tea that have been analyzed in this work (Table A1, Table A2, Table A3, Table A4, Table A5, Table A6, Table A7, Table A8, Table A9, Table A10 and Table A11).

**Table A1:** Results for the TGO sample fingerprint determination with indication of the retention time (RT) and abundance mean for every identified compound.

RT	Compound Name	Abundance Mean
0.745	Acetic acid, cyano-	168,502
0.830	2-Amino-4-dimethylaminomethylenepentanedinitrile	108,883
1.054	Ethane-1,2-diimine, N,N′-diamino-	109,436
1.065	Acetone	114,706
2.575	Butanoic acid, ethyl ester	3,839,625.5
3.264	Hexanal	722,081
4.235	9-Tetradecen-1-ol, acetate, (E)-	256,238
5.942	D-Limonene	13,781,429.5
6.678	2-Hexenal	213,594
6.993	N-(Trifluoroacetyl)-N,O,O′,O″-tetrakis(trimethylsilyl)norepinephrine	377,968
7.628	(+)-3-Carene, 10-(acetylmethyl)-	259,938
7.630	3(10)-Caren-4-ol, acetoacetic acid ester	498,827
8.286	1-Pentanol	208,402
8.518	Nonane, 5-(2-methylpropyl)-	239,091
9.647	Octanal	165,442.5
10.755	3-Ethyl-3-methylheptane	114,704
10.976	2-Heptenal, (Z)-	207,932.5
11.957	5-Hepten-2-one, 6-methyl-	623,348
14.761	Nonanal	581,017.5
15.175	Oxirane, [(tetradecyloxy)methyl]-	193,068
15.666	Oxalic acid, propyl undecyl ester	165,284
16.277	2-Undecenal, E-	121,169
16.306	Oxirane, 2,2′-(1,4-butanediyl)bis-	119,155
17.193	2-Furanmethanol, 5-ethenyltetrahydro-.alpha.,.alpha.,5-trimethyl-, cis-	1,799,498
17.647	Undecanol-4	291,287
17.678	3-Octanol, 3,6-dimethyl-	239,713
18.343	Ammonium acetate	1,079,115.5
18.768	2-Furanmethanol, 5-ethenyltetrahydro-.alpha.,.alpha.,5-trimethyl-, cis-	2,039,755
19.085	Octadecane, 1-chloro-	603,170
19.587	Acetic acid, hexyl ester	601,861
19.760	1,6-Heptadiene, 3,5-dimethyl-	80,227
20.519	Decanal	622,713
21.134	Benzaldehyde	94,325
22.424	3-Cyclohexene-1-ethanol, .beta.,4-dimethyl-	131,133
22.431	1-Cyclohexene-1-methanol, 4-(1-methylethenyl)-	140,779
22.670	3-Heptyne-2,6-dione, 5-methyl-5-(1-methylethyl)-	134,078
22.801	Cyclobutane, 1,2-bis(1-methylethenyl)-, trans-	214,018
22.816	1-Isopropenyl-3-propenylcyclopentane	189,887
23.354	Cyclohexanol, 2,2,6,6-tetramethyl-	1,107,971
23.360	Citronellyl butyrate	1,018,024
24.182	1,6-Octadien-3-ol, 3,7-dimethyl-, 2-aminobenzoate	6,824,657.5
24.495	1-Octanol	905,951.5
24.911	Cyclohexane, 1-ethenyl-1-methyl-2,4-bis(1-methylethenyl)-, [1S-(1.alpha.,2.beta.,4.beta.)]-	122,847.5
25.385	1,1-Dimethyl-4-methylenecyclohexane	277,392
25.417	Cyclohexanone, 2-methyl-5-(1-methylethenyl)-, trans-	243,765
27.500	Hotrienol	80,758
27.795	Carveol	545,775
27.797	Dispiro[2 .1.2.4]undecane, 8-methylene-	617,343
28.060	Acetophenone	82,331
28.301	Hexanoic acid, 6-bromo-	217,313
29.001	2-Cyclohexen-1-one, 2-methyl-5-(1-methylethyl)-, (S)-	532,374.5
30.125	Carveol	474,827
30.130	6-Isopropenyl-3-methoxymethoxy-3-methyl-cyclohexene	465,247
30.242	1-Nonanol	768,727.5
30.972	cis-p-Mentha-2,8-dien-1-ol	560,885
30.974	trans-p-mentha-1(7),8-dien-2-ol	662,768
31.523	.alpha.-Terpineol	1,098,969
32.232	(-)-Carvone	3,383,059
32.611	Undecane, 2-methyl-	74,437
32.624	Pentadecane	155,418
33.721	2H-Pyran-3-ol, 6-ethenyltetrahydro-2,2,6-trimethyl-	203,784
33.982	Carveol	450,597
34.200	Ethanone, 1-(3-methylphenyl)-	150,098
34.340	1-Cyclohexene-1-carboxaldehyde, 4-(1-methylethenyl)-	262,883
34.627	Decane, 1,1′-oxybis-	92,683
35.010	2H-Pyran-3-ol, 6-ethenyltetrahydro-2,2,6-trimethyl-	109,855
35.173	Cyclopropane, octyl-	181,727
35.176	Decane, 3-chloro-	131,903
35.319	6-Octen-1-ol, 3,7-dimethyl-, (R)-	158,561
35.332	Citronellal	169,686
35.896	p-Mentha-1(7),8-dien-2-ol	152,465
36.474	2-Cyclohexen-1-one, 3-methyl-6-(1-methylethenyl)-, (S)-	98,294
36.694	3-hydroxy-2-methyl-5-(prop-1-en-2-yl)cyclohexanone	250,595
37.280	2-Cyclohexen-1-ol, 2-methyl-5-(1-methylethenyl)-, acetate, (1R-cis)-	1,127,661
37.837	Heptanoic acid	1,468,435.5
38.200	trans-p-mentha-1(7),8-dien-2-ol	380,766
38.414	Benzyl-diseryl phosphate	162,123.5
38.840	(2R,4R)-p-Mentha-[1(7),8]-diene, 2-hydroperoxide	97,386
40.616	(-)-trans-Myrtanyl acatate	105,666
40.623	Myrcenylacetat	146,060
40.857	Cyclododeca-5,9-dien-1-ol, 2-methyl-, (Z,Z)-	317,443
40.859	(2R)-Bornane-10,2-sultam	219,341
41.850	Benzaldehyde, 4-methoxy-	233,353
43.263	Octanoic acid	191,580
43.436	Cyclohexanemethanol, 4-ethenyl-.alpha.,.alpha.,4-trimethyl-3-(1-methylethenyl)-, [1R-(1.alpha.,3.alpha.,4.beta.)]-	103,468
44.618	2-Pentadecanone, 6,10,14-trimethyl-	69,726
45.482	Dodecanoic acid, 3-hydroxy-	256,424
45.502	n-Hexadecanoic acid	335,278
47.527	2,6-Octadiene-1,8-diol, 2,6-dimethyl-	896,585.5
47.940	2(4H)-Benzofuranone, 5,6,7,7a-tetrahydro-4,4,7a-trimethyl-	344,852.5
50.351	Benzophenone	173,741.5
51.518	Vanillin	206,667.5

**Table A2:** Results for the SGP sample fingerprint determination with indication of the retention time (RT) and abundance mean for every identified compound.

RT	Compound Name	Abundance Mean
1.073	Cyclopropyl methyl carbinol	120,514
3.273	Hexanal	575,125.5
3.405	Methoxyacetic acid, tridecyl ester	68,993
3.430	3-Tridecene, (Z)-	68,920
4.183	4-Pentenal, 2-ethyl-	115,550
4.460	Sydnone, 3,3′-trimethylenedi-	22,564
5.214	.beta.-Myrcene	1,018,173.5
5.260	.beta.-Myrcene	619,254
5.490	Ethanol, 2-[2-(4-pyridyl)ethylamino]-	42,808
5.921	Cyclohexene, 4-ethenyl-1,4-dimethyl-	639,379
6.702	5-Aminoisoxazole	42,411
6.706	3-Hexenal, (Z)-	364,986
7.009	N-(Trifluoroacetyl)-N,O,O′,O″-tetrakis(trimethylsilyl)norepinephrine	118,904
7.601	.beta.-Ocimene	110,410
7.615	.alpha.-Pinene	433,550
8.170	3-Carene	119,228
8.215	Tricyclo[2.2.1.0(2,6)]heptane, 1,7,7-trimethyl-	156,509
8.229	.beta.-Ocimene	630,021
8.464	Dodecane, 4,6-dimethyl-	129,460
8.475	Undecane, 3,8-dimethyl-	507,030
10.385	trans,cis-2,6-Nonadienyl acetate	34,989
10.467	Tridecane, 6-methyl-	210,574
10.497	Nonane, 4,5-dimethyl-	37,215
10.734	Dodecane, 4,6-dimethyl-	338,715.5
11.000	3-Hepten-1-ol, (Z)-	226,106
11.151	3-Ethyl-3-methylheptane	239,089
11.425	cis-Aconitic anhydride	27,787
11.556	4-Heptafluorobutyroxytridecane	524,738
11.586	Nonane, 4,5-dimethyl-	150,122
11.930	5-Hepten-2-one, 6-methyl-	378,933
12.318	2-Isopropyl-5-methyl-1-heptanol	195,059
12.323	Oxalic acid, allyl octadecyl ester	89,397
12.380	l-Alanine, N-(cyclohexylcarbonyl)-, heptadecyl ester	37,359
14.688	Nonanal	113,348
15.129	1-Methoxy-3-hydroxymethylheptane	119,355
15.620	Heptadecane, 2,6,10,14-tetramethyl-	65,889
15.635	Tridecane	183,448
16.195	3-Undecene, (Z)-	63,096
16.220	2-Nonenal, (Z)-	147,528
16.460	2-(3-Bromopropyl)-[1,3]dioxolane	28,267
18.280	Ammonium acetate	187,763
18.330	Methoxyacetic acid, hexyl ester	48,206
18.390	1,4-Hexadiene, 5-methyl-	261,153
18.429	Cyclohexan-1,4,5-triol-3-one-1-carboxylic acid	619,199
18.661	Furfural	345,710
19.005	Methoxyacetic acid, 2-tridecyl ester	57,046
19.022	Nonane, 3-methyl-5-propyl-	417,744
19.055	Nonane, 4,5-dimethyl-	72,220
19.600	Butane, 1-(ethenyloxy)-	23,034
19.715	2,3-Hexadiene, 2-methyl-	388,031
20.635	2-Propyl-1-pentanol	135,168
21.037	Benzaldehyde	246,318
21.250	Undecane, 3,6-dimethyl-	147,124
21.466	R-Limonene	197,652
21.730	Pyrrolidine-2,4-dione	21,913
21.764	Dodecane, 2,6,11-trimethyl-	130,862
21.770	Decane, 2-methyl-	37,031
22.158	1,5-Heptadiene, 2,3,6-trimethyl-	635,307.5
23.267	6-Octen-1-ol, 3,7-dimethyl-, acetate	589,174
23.301	Cyclopropanemethanol, .alpha.,2-dimethyl-2-(4-methyl-3-pentenyl)-, [1.alpha.(R*),2.alpha.]-	1,041,462
23.660	N-[2,2,2-Trifluoro-1-(isopropylamino)-1-(trifluoromethyl)ethyl]isovaleramide	197,837
24.198	1,6,10-Dodecatrien-3-ol, 3,7,11-trimethyl-	18,728,058
24.205	1-Bromo-3,7-dimethyl-2,6-octadiene	31,337,534
24.306	Linalyl acetate	10,646,193
25.300	3-hydroxy-2-methyl-5-(prop-1-en-2-yl)cyclohexanone	105,570
26.919	Oxalic acid, ethyl neopentyl ester	276,546
26.940	Undecane, 2-methyl-	248,945
27.153	Ethanol, 2-(2-ethoxyethoxy)-	337,777
27.405	1,5-Heptadiene, 2,5-dimethyl-3-methylene-	127,621
27.590	Cyclohexane, 1R-acetamido-2,3-cis-epoxy-4-cis-formyloxy-	41,150
27.948	Acetophenone	217,637
28.322	d-Menthol	135,041
30.080	1-Pentene, 5-(2,2-dimethylcyclopropyl)-2-methyl-4-methylene-	495,428
30.086	(-)-cis-Myrtanol	169,778
30.505	Acetic acid, cyano-	116,530
30.842	Cyclobutane, 1,2-bis(1-methylethenyl)-, trans-	607,249.5
31.440	.alpha.-Terpineol	3,145,562.5
32.278	Heptafluorobutanoic acid, 2-(1-adamantyl)ethyl ester	611,947
32.279	2,4-Methano-1H-indene, 4-chlorooctahydro-	186,219
32.657	.beta.-Bisabolene	192,658.5
32.958	2,6-Octadienal, 3,7-dimethyl-, (E)-	342,345.5
33.300	1-Bromo-3,7-dimethyl-2,6-octadiene	1,139,696
33.680	2H-Pyran-3-ol, 6-ethenyltetrahydro-2,2,6-trimethyl-	138,117
34.317	Dispiro[4.2.4.2]tetradecane	118,050
34.327	Cis-8-ethyl-exo-tricyclo[5.2.1.0(2.6)]decane	66,678
34.660	1-Bromo-3,7-dimethyl-2,6-octadiene	1,387,273
36.273	5-Hepten-1-ol, 2-ethenyl-6-methyl-	386,406
36.587	Anethole	232,191
37.173	Lanceol, cis	198,363
37.177	Ionone	218,275
37.344	1,4-Methanobenzocyclodecene, 1,2,3,4,4a,5,8,9,12,12a-decahydro-	517,247.3
37.793	1,2,4-Trioxolane, 3,5-dipropyl-	599,431
37.811	Octane, 1-azido-	718,189
37.900	2,6,10-Dodecatrien-1-ol, 3,7,11-trimethyl-, (Z,E)-	332,897
38.200	2H-Azepin-2-one, hexahydro-4-methyl-	111,168
38.205	1-Undecene, 5-methyl-	150,120
38.453	2-Methylvaleroyl chloride	184,447
38.730	Geranyl acetate, 2,3-epoxy-	139,279
39.503	2-Dimethyl(trimethylsilyl)silyloxytridecane	37,938
39.686	2-Butanone, 4-(2,6,6-trimethyl-2-cyclohexen-1-ylidene)-	212,113.5
40.236	3-Decyn-2-ol	108,829
40.269	3-Tetradecyn-1-ol	127,313
40.807	Cyclododeca-5,9-dien-1-ol, 2-methyl-, (Z,Z)-	151,636
41.095	1,5,5-Trimethyl-6-methylene-cyclohexene	370,901
41.107	1,3,6-Heptatriene, 2,5,6-trimethyl-	312,389
44.479	1-Heptyn-6-one	88,109
44.600	9-Decen-2-one, 5-methylene-	126,372
44.613	1-Heptyn-6-one	278,250
44.807	10-Undecyn-1-ol	104,806
44.819	Cyclopropane, hexylidene-	158,800
46.771	Acetic acid, cyano-	106,057
47.899	2(4H)-Benzofuranone, 5,6,7,7a-tetrahydro-4,4,7a-trimethyl-	484,359.5
57.725	Imidodicarbonic acid, diethyl ester	132,177

**Table A3:** Results for the SGL sample fingerprint determination with indication of the retention time (RT) and abundance mean for every identified compound.

RT	Compound Name	Abundance Mean
0.879	Acetic acid, cyano-	56,772
1.040	Pentanal, 2,4-dimethyl-	57,277
1.071	1-Propen-2-ol, formate	167,675
2.285	.alpha.-Pinene	170,241.5
2.328	1,3,6-Octatriene, 3,7-dimethyl-, (Z)-	363,519
2.385	(R)-(+)-Citronellic acid	85,004
2.460	3-Undecen-1-yne, (E)-	7687
3.225	Hexanal	28,897
3.965	Methanone, 1,3-dithian-2-ylphenyl-	64,181
6.013	D-Limonene	44,500,934
6.715	4-Pentenal, 2-methyl-	50,578
7.642	.gamma.-Terpinene	6,561,102
8.201	.beta.-Ocimene	328,361
8.607	Benzene, 1-methyl-3-(1-methylethyl)-	2,692,739.5
9.068	2-Carene	3,351,302
9.078	(+)-4-Carene	3,540,274
10.465	Octane, 2,3,3-trimethyl-	44,003
10.752	Undecane, 3,8-dimethyl-	74,779
11.947	5-Hepten-2-one, 6-methyl-	273,176.5
13.846	1-Octanol, 3,7-dimethyl-, (S)-	180,675
14.722	Nonanal	92,985
15.668	Tridecane	139,294
16.709	Benzene, 2-ethenyl-1,3-dimethyl-	191,674
16.740	o-Isopropenyltoluene	194,054
18.399	Cyclohexan-1,4,5-triol-3-one-1-carboxylic acid	361,809
18.408	Glycidol	717,548
18.670	Furfural	621,339
18.970	Copaene	551,737
18.986	.alfa.-Copaene	507,260
19.180	Decane, 5-propyl-	88,193
19.434	6-Octenal, 3,7-dimethyl-, (R)-	837,850
19.699	3-Octen-2-ol, (Z)-	451,255
19.722	Cyclopropane, trimethylmethylene-	866,370
20.190	E-1,5,9-Decatriene	64,911
20.665	1-Methoxy-3-hydroxymethylheptane	126,181
21.051	1,2,4-Trioxolane, 3-methyl-5-phenyl-	59,002
21.430	Octane, 5-ethyl-2-methyl-	168,365
21.683	(Z,Z)-.alpha.-Farnesene	330,622
21.813	Nonane, 5-(2-methylpropyl)-	161,847
22.189	3-Ethyl-1,5-octadiene	224,260
23.256	trans-p-mentha-1(7),8-dien-2-ol	842,313
23.272	cis-p-mentha-1(7),8-dien-2-ol	736,608
24.139	1-Bromo-3,7-dimethyl-2,6-octadiene	8,422,457
24.146	.beta.-Myrcene	7,426,440
24.450	Caryophyllene	2,412,339
24.803	Cyclohexane, 1-ethenyl-1-methyl-2,4-bis(1-methylethenyl)-	1,661,763
24.828	cis-.alpha.-Bisabolene	1,997,395
25.130	Dodecanal	957,818
25.148	trans-2-Dodecen-1-ol, trifluoroacetate	522,026
26.458	Cyclohexane, 4-methyl-2-methylene-1-(1-methylethylidene)-	236,924
27.055	Pentadecane	615,120
27.122	Heptadecane, 2,6,10,14-tetramethyl-	369,320
27.928	3-Tetradecyn-1-ol	964,333
27.958	trans-2-Dodecen-1-ol, trifluoroacetate	840,881
28.359	1,4,7,-Cycloundecatriene, 1,5,9,9-tetramethyl-, Z,Z,Z-	245,910
28.761	2,2-Dimethylpropanoic acid, 2-adamantyl ester	255,092
29.215	Cycloisolongifolene	97,234
30.355	2,6-Octadienal, 3,7-dimethyl-, (E)-	51,100,548.5
31.498	.alpha.-Terpineol	8,024,237
32.200	(-)-Carvone	653,884
32.388	Dispiro[4.2.4.2]tetradecane	107,256
32.450	3-Adamantanecarboxylic acid, phenyl ester	701,488
33.255	2,6-Octadienal, 3,7-dimethyl-, (E)-	71,369,488.5
33.958	Carveol	117,489
33.989	Cycloheptane, 1,3,5-tris(methylene)-	157,810
34.465	Naphthalene, 1,2,3,4,4a,5,6,7-octahydro-4a-methyl-	102,571
34.475	1H-3a,7-Methanoazulene, 2,3,6,7,8,8a-hexahydro-1,4,9,9-tetramethyl-, (1.alpha.,3a.alpha.,7.alpha.,8a.beta.)-	90,857
34.689	1-Bromo-3,7-dimethyl-2,6-octadiene	594,352
35.305	7-Octen-1-ol, 3,7-dimethyl-, (S)-	473,977
36.040	Z,Z,Z-4,6,9-Nonadecatriene	914,01
36.294	5-Hepten-1-ol, 2-ethenyl-6-methyl-	1,012,776
36.612	Anethole	319,573
37.369	1,4-Methanobenzocyclodecene, 1,2,3,4,4a,5,8,9,12,12a-decahydro-	867,511.5
37.929	5-Hepten-1-ol, 2-ethenyl-6-methyl-	1,881,320
37.939	1-Bromo-3,7-dimethyl-2,6-octadiene	2,378,768
38.473	1-Hexene, 3,4,5-trimethyl-	2,227,084.5
39.175	2-Bromosebacic acid, bis(trimethylsilyl) ester	120,406
39.715	2-Butanone, 4-(2,6,6-trimethyl-2-cyclohexen-1-ylidene)-	172,929
40.354	Caryophyllene	167,562
41.044	2-Butanone, 4-(2,6,6-trimethyl-1-cyclohexen-1-yl)-	182,625
41.120	11,11-Dimethyl-spiro[2,9]dodeca-3,7-dien	156,106
41.352	5-Hepten-2-one, 6-methyl-	57,635
41.943	2-Cyclohexen-1-one, 6-(1-hydroxy-1-methylethyl)-3-methyl-	71,111
43.753	1-Nitro-2-propanone	369,292
44.620	1-Heptyn-6-one	806,489
44.628	Methyl Isobutyl Ketone	840,137
44.750	trans,trans-2,6-Dimethyl-2,6-octadiene-1,8-diol	174,272
47.500	Cyclopropaneoctanoic acid, 2-[[2-[(2-ethylcyclopropyl)methyl]cyclopropyl]methyl]-, methyl ester	105,937
47.515	Tetraacetyl-d-xylonic nitrile	34,187
47.917	2(4H)-Benzofuranone, 5,6,7,7a-tetrahydro-4,4,7a-trimethyl-	481,958
48.378	Cycloheptane, 4-methylene-1-methyl-2-(2-methyl-1-propen-1-yl)-1-vinyl-	99,068
52.790	1-Propanamine, N-nitro-	37,512
52.992	1-Butanol	59,088

**Table A4:** Results for the SGM sample fingerprint determination with indication of the retention time (RT) and abundance mean for every identified compound.

RT	Compound Name	Abundance Mean
0.018	Acetic acid, cyano-	63,013
0.825	Ethanol, 2-(vinyloxy)-	60,490
1.055	Hydroperoxide, 1-methylethyl	85,713
1.120	Ether, 2-chloro-1-propyl isopropyl	63,783
3.259	Hexanal	352,944
3.350	Glutaraldehyde	150,508
3.355	3-Penten-2-ol	315,320
3.440	1,2,15-Pentadecanetriol	149,001
3.490	Trifluoromethanesulfonyl imidazole	146,098
3.540	Acetic acid, cyano-	63,215
4.239	9-Tetradecen-1-ol, acetate, (E)-	81,718
5.184	2-Octyn-1-ol	825,999
5.211	.beta.-Myrcene	501,590
5.665	2-Heptanone	127,653
5.912	D-Limonene	201,138
5.918	Cyclohexene, 4-ethenyl-1,4-dimethyl-	109,577
6.190	3-Pyridinecarbonitrile, 4-(methoxymethyl)-6-methyl-2-(2-propenyloxy)-	64,607
6.706	2-Hexenal	151,942
6.714	(1-Allylcyclopropyl)methanol	133,747
6.957	N-(Trifluoroacetyl)-N,O,O′,O″-tetrakis(trimethylsilyl)norepinephrine	55,876
7.511	Furfuryl heptanoate	99,402
7.556	.alpha.-Pinene	97,319
7.630	4-Terpinenyl acetate	61,282
8.220	.beta.-Ocimene	235,507
8.486	Undecane, 3,8-dimethyl-	261,838
8.499	Dodecane, 4,6-dimethyl-	334,363
8.784	Octane, 2,3,3-trimethyl-	140,125
9.045	(+)-4-Carene	55,258
10.420	trans-.beta.-Terpinyl pentanoate	98,048
10.485	Methoxyacetic acid, 2-methylpropyl ester	72,844
10.729	Undecane, 2-methyl-	153,807
10.739	Nonane, 5-(2-methylpropyl)-	210,592
10.963	Butanamide, 3-cyclohexylamino-4-hydroxy-N-cyclohexyl-	233,226
10.972	Z-1,8-Dodecadiene	197,182
11.190	Nonane, 1-iodo-	843,66
11.265	2,4-Pentanedione, 3-ethyl-	65,334
11.453	1-Pentene, 5-chloro-	326,317.5
11.595	Undecane, 2,8-dimethyl-	101,042
11.910	5-Hepten-2-one, 6-methyl-	361,742.5
12.318	Trichloroacetic acid, tridecyl ester	59,185
14.315	Diazene, dicyclohexyl-, 1,2-dioxide	61,943
14.698	Nonanal	167,658
15.146	2-Decen-1-ol	146,286
15.591	Heptadecane, 2,6,10,14-tetramethyl-	59,042
15.627	Dodecane, 2-methyl-	75,909
16.206	5-Tridecene, (Z)-	68,987
16.267	2-Octyn-1-ol	71,981
18.250	2,3-Epoxybutane	411,295
18.445	6-Nonenal, (Z)-	530,076
18.452	Imidazole, 2-amino-5-[(2-carboxy)vinyl]-	245,253
18.654	Furfural	266,284.5
19.036	Nonane, 5-(1-methylpropyl)-	138,397
19.039	Nonane, 3-methyl-5-propyl-	243,464
19.698	2,3-Hexadiene, 2-methyl-	277,170
20.606	1-Pentanol, 2-ethyl-4-methyl-	129,402
20.995	Benzaldehyde	165,013
21.054	1-Benzamido-N-benzyl-1-[.alpha.-(2-pyridylthio)benzylidene]acetamide	119,058
21.205	Nonane, 4,5-dimethyl-	83,886
21.426	R-Limonene	119,271
21.758	Dodecane, 4-methyl-	78,992
22.139	1,5-Heptadiene, 2,3,6-trimethyl-	209,903
23.194	Heptanal, 2-methyl-	278,302
23.262	2-Octen-1-ol, 3,7-dimethyl-	450,603
23.615	2-Heptafluorobutyroxydodecane	70,417
24.087	1-Bromo-3,7-dimethyl-2,6-octadiene	9,842,404
25.104	Oxirane, decyl-	134,290
25.211	Dihydrocarvyl acetate	90,710
26.920	Tridecane	151,803
26.937	Pentadecane	175,135
27.128	Ethanol, 2-(2-ethoxyethoxy)-	248,708.5
27.364	Dispiro[2.0.2.5]undecane, 8-methylene-	120,439
27.894	S-Benzoyl-N-(O-hydroxybenzylidene)thiohydroxylamine	87,628
28.021	2-Decene, (Z)-	71,624
28.284	Cyclohexanol, 5-methyl-2-(1-methylethyl)-, [1S-(1.alpha.,2.alpha.,5.beta.)]-	60,158
30.084	Longipinene epoxide	200,527
30.135	cis-p-mentha-1(7),8-dien-2-ol	89,537
30.355	Valeric acid, 3-tridecyl ester	80,075
30.565	Acetaldoxime	68,044
30.803	Cyclobutane, 1,2-bis(1-methylethenyl)-, trans-	211,358.5
31.404	.alpha.-Terpineol	762,821.5
32.214	2,2-Dimethylpropanoic acid, 2-adamantyl ester	60,028
32.296	2,4-Methano-1H-indene, 4-chlorooctahydro-	118,582
32.590	cis-sesquisabinene hydrate	117,388
32.929	2,6-Octadien-1-ol, 2,7-dimethyl-	89,883.5
33.278	1-Bromo-3,7-dimethyl-2,6-octadiene	288,750
33.284	4-Hexen-1-ol, 5-methyl-2-(1-methylethenyl)-, acetate	343,381
33.572	Cyclopentane, 1-ethyl-3-methyl-, trans-	52,106
33.967	Pentanal	185,683
34.280	Cyclododeca-5,9-dien-1-ol, 2-methyl-, (Z,Z)-	84,052
34.310	Cis-8-ethyl-exo-tricyclo[5.2.1.0(2.6)]decane	65,264
34.643	2,6-Octadien-1-ol, 2,7-dimethyl-	492,969
34.648	.beta.-Myrcene	508,110
36.243	5-Hepten-1-ol, 2-ethenyl-6-methyl-	133,316
36.577	Anethole	199,512
37.160	Andrographolide	77,139
37.584	1,4-Methanobenzocyclodecene, 1,2,3,4,4a,5,8,9,12,12a-decahydro-	696,692.5
37.779	Hexanal	1,321,771
37.793	1,2,4-Trioxolane, 3,5-dipropyl-	219,894
37.875	.beta.-Myrcene	172,198
38.180	2,5-Pyrrolidinedione, 1-ethyl-	131,028
38.429	2-Butanone, 3-methyl-1-phenyl-	153,622
38.435	4-Ethyl-1-hexyn-3-ol	192,370
38.771	Cyclopropane, 1-(1′-propenyl)-2-hydroxymethyl-	75,503
39.686	Acetic acid, 6,6-dimethyl-2-methylene-7-(3-oxobutylidene)oxepan-3-ylmethyl ester	61,416
40.818	3-Tridecen-1-yne, (Z)-	69,714
40.819	9,12,15-Octadecatrienal	97,882
41.011	2-Butanone, 4-(2,6,6-trimethyl-1-cyclohexen-1-yl)-	102,986
41.093	cis-sesquisabinene hydrate	224,962
44.166	Adenine-9-propanoic acid, alpha.-t-butoxycarbonylamino-	102,494
44.463	1-Heptyn-6-one	98,989
44.469	Methyl Isobutyl Ketone	63,708
44.595	9-Decen-2-one, 5-methylene-	143,812
45.240	Propanal, 2-methyl-, 2-propenylhydrazone	52,896
45.470	Cyclopentaneundecanoic acid	64,619
47.888	2(4H)-Benzofuranone, 5,6,7,7a-tetrahydro-4,4,7a-trimethyl-	283,652

**Table A5:** Results for the TBV sample fingerprint determination with indication of the retention time (RT) and abundance mean for every identified compound.

RT	Compound Name	Abundance Mean
0.730	Acetic acid, cyano-	94,784
0.815	Propane	115,993
0.881	N-(2-Methylacryloyl)imidazola	114,248
1.040	Allyl acetate	49,897
1.081	2-Hexanone, 4-hydroxy-5-methyl-3-propyl-	221,553
1.425	Pentan-2-ol, 1-tert-buthylamino-4-methyl-	37,600
1.438	Butanal, 2-methyl-	100,607
1.490	Butanal, 3-methyl-	84,716
1.947	3-Aminopyrrolidine	207,498
1.951	1,3-Dioxane-4,6-dione, 2,2-dimethyl-	225,463
2.030	Acetamide, N-[2-(4-methylphenoxy)ethyl]-	65,728
2.045	Azacyclodecan-5-ol	45,434
3.284	Hexanal	1,398,458.5
3.655	Triallyl phosphate	23,541
5.575	Acetonitrile, bromo-	26,297
5.675	Propane, 2-(ethenyloxy)-	24,723
5.730	2,3-Anhydro-d-galactosan	30,652
5.740	Heptanal	147,257
5.765	Butanal, 3-methyl-	149,962
5.953	Tetrahydropyrrolo[1,2-a]azetidin-2-one	193,616
5.957	Murexide	90,456
6.030	5-[2-Thienyl]hydantoin	77,334
6.702	2-Hexenal	676,577
6.985	N-(Trifluoroacetyl)-N,O,O′,O″-tetrakis(trimethylsilyl)norepinephrine	706,731
8.505	Dodecane, 4-methyl-	90,410
9.655	Octanal	66,979
9.658	1,6-Anhydro-3,4-dideoxy-.beta.-D-manno-hexapyranose	54,785
11.959	5-Hepten-2-one, 6-methyl-	262,786
14.740	Nonanal	373,934.5
15.147	Ethanol, 2-butoxy-	138,163
15.165	2,5-Dimethyl-1-hepten-4-ol	121,419
18.282	Ammonium acetate	2,141,214
18.701	Furfural	1,136,801
19.103	Nonane, 5-(2-methylpropyl)-	246,297
20.714	1-Hexanol, 2-ethyl-	75,528
21.122	Benzaldehyde	4,085,838.5
21.846	Undecane, 3,7-dimethyl-	316,312
22.135	Cycloheptano[d]imidazolidine, 1,3-dihydroxy-2-methyl-	103,470
23.290	Propanoic acid	200,796.5
24.141	5-Hepten-1-ol, 2-ethenyl-6-methyl-	197,773
24.456	1H-Pyrazole, 1,3,5-trimethyl-	783,099
24.467	2-Furancarboxaldehyde, 5-methyl-	686,396
26.464	Benzenemethanol, .alpha.-(1-ethenylpentyl)-.alpha.-methyl-	96,576
27.076	Undecane, 2-methyl-	255,153
27.700	Benzaldehyde, 2-methyl-	129,230.5
28.036	Acetophenone	69,030
30.240	Pent-3-en-2-one, 4-methyl-, oxime	127,754
30.255	4-Methoxy-2,3-dimethyl-2,3-dihydroazete	103,964
30.487	2(3H)-Furanone, 5-ethyldihydro-	554,528.5
32.606	2,3-Dimethyldodecane	55,910
32.606	Tetradecane, 2-methyl-	71,677
33.011	Propanoic acid, 3-hydroxy-2-[2-{[benzyloxy)carbonyl]amino}acetyl)amino]	43,642
34.024	Pentanoic acid	72,953.5
34.312	Methyl salicylate	34,235
35.313	2(3H)-Furanone, dihydro-5-propyl-	3,103,701.5
36.641	1-Octadecanesulphonyl chloride	85,226.5
37.648	1,4-Methanobenzocyclodecene, 1,2,3,4,4a,5,8,9,12,12a-decahydro-	91,628.5
37.840	Hexanoic acid	887,087.5
37.928	Phenol, 2-methoxy-	624,495
38.239	2,5-Pyrrolidinedione, 1-ethyl-	345,675.5
38.411	Benzyl-diseryl phosphate	193,527
38.413	Benzene, [(2-propenyloxy)methyl]-	326,206
38.993	2(3H)-Furanone, 5-butyldihydro-	567,940.5
39.296	Phenylethyl Alcohol	81,672
40.533	Maltol	3,012,548.5
40.765	Heptanoic acid	213,937
40.869	2-Pentenenitrile, 4,4-dimethyl-	282,191
40.879	7-Nonynoic acid	362,88
41.025	2-Hexenoic acid	206,927
41.060	1H-Azepine, hexahydro-1-nitroso-	33,836
41.175	1-Dodecene	170,528
41.180	E-11,13-Tetradecadien-1-ol	25,182
41.435	Furan, 2,2′-[oxybis(methylene)]bis-	124,175
41.845	Benzaldehyde, 4-methoxy-	2,417,723
42.050	1H-Pyrrole-2-carboxaldehyde	73,396
42.195	Benzene, (2-methyl-1-methylenebutyl)-	90,494
42.201	Cinnamaldehyde, (E)-	126,790
42.348	Benzene, 1,4-dimethoxy-2-methyl-	85,105
43.259	Octanoic acid	731,575
44.420	4-Acetylanisole	102,679
44.425	3-Methoxyacetophenone	138,444
44.526	2(3H)-Furanone, 5-hexyldihydro-	597,573
44.745	Ethanol, 2-phenoxy-	39,625
44.909	1,3,5-Cycloheptatriene, 1-methoxy-	58,650.5
45.506	Nonanoic acid	69,740.5
46.205	Piperonal	87,421.5
46.704	2-n-Butyl furan	23,192
47.458	Benzenemethanol, 4-methoxy-	421,101.5
47.590	n-Decanoic acid	300,368
47.938	2(4H)-Benzofuranone, 5,6,7,7a-tetrahydro-4,4,7a-trimethyl-	278,448.5
48.989	.gamma.-Dodecalactone	258,188
49.980	Benzoic acid	62,533
51.376	1,2-Benzenedicarboxylic acid, dihexyl ester	26,249
51.531	Vanillin	8,287,611.5
54.111	3-Hydroxy-4-methoxybenzyl alcohol	51,514

**Table A6:** Results for the EBP sample fingerprint determination with indication of the retention time (RT) and abundance mean for every identified compound.

RT	Compound Name	Abundance Mean
0.189	Carbamic acid, (cyanoacetyl)-, ethyl ester	40,219
0.690	Acetic acid, cyano-	71,328
0.859	4-Heptanone, dimethylhydrazone	68,187
0.931	1-(4-Acetamidoanilino)-3,7-dimethylbenzo[4,5]imidazo[1,2-a]pyridine-4-carbonitrile	42,303
0.980	Sulfide, methyl 1-methyl-2-butenyl	44,455
1.390	4H,8H-[1,2,4]Triazino[3,4-b][1,3,4]thiadiazin-4-one, 7-amino-3-methyl-	48,156
1.430	Butanal, 3-methyl-	54,247
1.504	Acetic acid, cyano-	24,296
1.547	4-Penten-2-ol, 4-methyl-	36,478
1.719	2-Butyne-1,4-diol bis(.beta.-hydroxyethyl ether)	29,900
2.525	3-Hexenoic acid, ethyl ester, (Z)-	21,487
2.599	dl-Ornithine	27,137
2.618	Thiocyanic acid, 5-amino-3-methyl-4-isoxazolyl ester	22,308
2.740	5-Ethyl-2-methyl-pyridin-4-amine	27,062
3.220	Hexanal	95,779
3.273	Hexanal	32,777
3.295	1,3-Dioxane-4,6-dione, 5,5-dimethyl-2-(1-methylethylidene)-	36,361
3.308	Butane, 1-(ethenyloxy)-	82,998
3.335	1-Propene, 3-methoxy-	92,869
3.400	5-Aminoisoxazole	93,919
3.420	cis-Aconitic anhydride	33,724
3.435	2-Propanamine, N-ethyl-N-nitroso-	24,872
3.460	Tris(aziridinomethyl)hydrazine	24,606
3.504	Butane, 1-(ethenyloxy)-	22,708
3.505	4,5-Dicarboxy-1,2,3-triazole	23,452
5.125	Formic acid, 1,1-dimethylethyl ester	23,031
5.150	1,2,4-Triazol-5-acetic acid, 3-amino-	24,912
5.212	Ethanediamide, N-(1-methylpropyl)-N′-(3-pyridinylmethyl)-	21,740
5.225	cis-Aconitic anhydride	26,678
5.330	(+)-2-Carene, 4-.alpha.-isopropenyl-	21,260
5.740	3-(4-Methyl-piperazin-1-yl)-N-(4-trifluoromethoxy-phenyl)-propionamide	25,237
6.677	Allyl methallyl ether	54,578
6.974	N-(Trifluoroacetyl)-N,O,O′,O″-tetrakis(trimethylsilyl)norepinephrine	69,040
7.040	N-Methyladrenaline, tri-TMS	28,857
7.503	Cyclopropanecarboxylic acid	21,350
8.163	Chlorocarbonyl t-butoxy sulfide	30,269
8.186	3-Buten-2-ol, 3-methyl-	26,893
8.455	3-Ethyl-3-methylheptane	66,659
9.740	1,4-Dioxa-2-decalone	28,660
10.738	Decane, 3,7-dimethyl-	46,542
11.560	2-Bromononane	23,532
11.905	2-Ethyl-3-vinyloxirane	23,682
11.925	2-Pentenal, 2,4,4-trimethyl-	28,175
13.315	Cyclohexanemethanol, .alpha.-ethyl-	31,869
14.310	6-Chloro-2,4-dihydroxy-1,3-dimethylpyrimidine	21,426
14.469	Methyl piperidin-4-carboxylate	21,730
14.610	Pyrrolizidine-3-one-5-ol, ethyl ether	27,717
14.672	Nonanal	61,648
14.690	p-Nitro carbanilic acid, n-heptyl ester	23,421
14.785	3,4,4,-Trimethyl-1-pentyn-3-ol	24,211
14.834	3,3-Dimethyl-2-hydroxy-2-phenylthiomorpholine	28,086
15.047	Cyclohexane, 1,2-bis(t-butoxycarbonylmethoxy)-	27,138
17.085	Cyanamide, N-allyl-N-[2-(2-hydroxy-2-methylpropyl)-3,3-dimethylcyclopropyl]methyl-	34,017
17.175	8.beta.-17.alpha.-Dihydroxy-desoxycorticosterone	27,911
17.505	1,2,4-Triazole, 4-[N-(2-hydroxyethyl)-N-nitro]amino-	22,875
17.557	4-Heptanol, 2-methyl-	43,238
18.316	Ammonium acetate	46,413
18.370	1,2,4-Trioxolane, 3,5-dipropyl-	46,649
18.566	1,2-Cyclobutanedicarboxylic acid, 3-methyl-, dimethyl ester	26,028
18.639	2H-Pyran, 2-(3-butynyloxy)tetrahydro-	33,812
18.666	trans-2,7-Dimethyl-3,6-octadien-2-ol	41,759
19.001	5-Ethyl-4-tridecanone	32,467
19.037	Tetradecane, 5-methyl-	27,346
19.055	1-Butanol, 3-methyl-, nitrate	30,550
20.405	Propenone, 3-dimethylamino-1-[3-(3-dimethylaminoacryloyl)-2,6-dihydroxyphenyl]-	22,794
20.461	Sulfide, di(1,3-butadienyl)-	28,315
20.545	5,6,6-Trimethyl-hept-3-yne-2,5-diol	26,021
20.579	4-Hexen-2-one, 3-methyl-	29,025
20.582	1-Decene, 2,4-dimethyl-	45,611
20.970	3-tert-Butyl-5-chloro-2-hydroxybenzophenone	26,089
21.017	1-Benzamido-N-benzyl-1-[.alpha.-(2-pyridylthio)benzylidene]acetamide	43,002
21.020	Bis[4-acetamidophenylsulfonyl]phenyl methane	43,423
21.105	1,2,5-Oxadiazol-3-amine, N-cyclopropyl-4-[5-(trichloromethyl)-1,2,4-oxadiazol-3-yl]-	31,391
21.172	Oxalic acid, allyl decyl ester	48,664
21.714	5-Hydroxy-2,4,4-trimethyl-cyclopentane-1,3-dione	28,464
21.726	Tetradecane, 4-ethyl-	49,899
22.143	3-Ethyl-1,5-octadiene	65,512
23.217	2-Norbornanone, 6-chloro-3,3-dimethyl-, exo-	32,271
23.230	1,2-Dihydrolinalool	82,631
23.265	2-Propenyl-3-vinyloxirane	35,236
24.021	2,6-Octadien-1-ol, 2,7-dimethyl-	827,705
24.036	Linalyl acetate	886,873
24.803	2H-Pyran, 2-(3-butynyloxy)tetrahydro-	25,758
26.907	Malonic acid, bis(2-trimethylsilylethyl ester	52,354
27.985	1,8-Dichlorooctane	25,086
28.235	Methylene asparagine	26,750
28.269	2-Pyrrolidinone, 1-methyl-	85,192
28.271	3,3,3-Trifluoro-N-(2-fluorophenyl)-2-(trifluoromethyl)propionamide	31,186
30.675	Oxalic acid, allyl nonyl ester	26,882
31.325	.alpha.-Terpineol	63,064.5
31.374	3-Methyl-2-methylene-5-oxopyrrolidine-3-carbonitrile	31,392
31.400	trans-2,7-Dimethyl-3,6-octadien-2-ol	26,447
33.244	trans,cis-2,6-Nonadien-1-ol	25,713
33.895	Propanedioic acid, propyl-	35,831
34.580	5-(3,7-Dimethylocta-2,6-dienyl)-4-methyl-2,3-dihydrothiophene 1,1-dioxide	22,011
34.904	2H-Pyran-3-ol, 6-ethenyltetrahydro-2,2,6-trimethyl-	25,268
36.573	Allyl heptanoate	22,998
37.752	Heptanoic acid	56,211.5
37.880	.beta.-Myrcene	29,088
38.160	2,5-Pyrrolidinedione, 1-ethyl-	62,446
38.341	Benzene, [(2-propenyloxy)methyl]-	72,902
38.344	Benzyl alcohol	84,220
39.227	Phenylethyl Alcohol	46,827
40.794	Ethanone, 1-(1H-pyrrol-2-yl)-	29,239.5
42.939	2-Butenediamide, 2-methyl-, (E)-	24,382
45.070	Benzylamine, N-(3-chloro-2,2-dimethyl-1-phenylpropylidene)-	25,983
45.373	Cyclopropanecarboxylic acid, cyclohexylmethyl ester	23,678
46.290	2-Amino-4-dimethylaminomethylenepentanedinitrile	30,668
47.859	2(4H)-Benzofuranone, 5,6,7,7a-tetrahydro-4,4,7a-trimethyl-	35,368
48.860	2H-Pyran-2-one, 5,6-dihydro-4-(2-methyl-3-methylene-1-buten-4-yl)-	76,496
48.921	3H-1,2,4-Triazole-3-thione, 2,4-dihydro-4-phenyl-	36,542
49.912	4-Piperidinepropanoic acid, 1-benzoyl-3-(2-chloroethyl)-, ethyl ester	44,931
52.555	1,2,3-Butanetriol	25,538
53.001	Oxetane, 2-methyl-4-propyl-	30,545
56.669	2,5-Furandione, dihydro-3-methylene-	29,961

**Table A7:** Results for the EBL sample fingerprint determination with indication of the retention time (RT) and abundance mean for every identified compound.

RT	Compound Name	Abundance Mean
0.886	p-Dioxane, methylene-	60,857
2.270	Hexanamide, 6-(2-oxocyclopentyl)-N-phenyl-	97,276
2.296	2-Hydroxy-3-pyrazin-2-ylacrylic acid	53,348
2.410	1,1′-(4-Methyl-1,3-phenylene)bis[3-(5-benzyl-1,3,4-thiadiazol-2-yl)urea]	66,587
3.230	Hexanal	125,407
4.240	Cyclohexene, 1-methyl-4-(1-methylethyl)-	415,553
5.165	.beta.-Myrcene	932,205.5
6.028	D-Limonene	20,772,492
6.083	Cyclobutane, 1,2-bis(1-methylethenyl)-, trans-	28,705,656
6.680	2-Hexenal	350,397
6.970	4-Methylcatechol, bis(trimethylsilyl) ether	99,509
7.608	.gamma.-Terpinene	2,668,285.5
8.218	3-Nonen-1-yne, (Z)-	185,302
8.577	Benzene, 1-methyl-3-(1-methylethyl)-	1,380,619
9.032	(+)-4-Carene	369,547.5
10.477	Decane, 2,4-dimethyl-	59,486
10.489	Undecane, 2,7-dimethyl-	147,641
11.408	Cyclopropaneethanol	87,457.5
11.920	1-Hepten-6-one, 2-methyl-	216,541
13.323	1-Butanol, 3-methoxy-	82,569
14.474	3-Hexen-1-ol	72,691
14.653	Nonanal	227,838
15.801	2-Penten-1-ol, 4-methyl-	123,248
17.298	2,6-Octadiene-1,8-diol, 2,6-dimethyl-	143,284
18.203	Ammonium acetate	594,590.5
18.619	Pyrazole, 1,4-dimethyl-	146,710
18.640	Furfural	251,584
18.926	Copaene	187,151
19.482	Acetic acid, hexyl ester	223,391
20.390	Octane, 1-azido-	48,281
20.413	Decanal	82,253
21.002	Benzaldehyde	147,588
22.121	1,6-Heptadiene, 2,5,5-trimethyl-	81,076
22.161	1,5-Heptadiene, 2,3,6-trimethyl-	245,420
23.162	5-Nonenoic acid, methyl ester	231,994
23.204	1,2-Dihydrolinalool	226,441
23.628	Cyclohexanol, 5-methyl-2-(1-methylethyl)-, acetate, (1.alpha.,2.beta.,5.beta.)-	54,549
23.656	Menthyl acetate	82,100
24.079	Linalyl acetate	3,330,821
24.326	1,1-Cyclopropanedicarbonitrile, 2-butyl-2-methyl-	176,787
24.694	trans-.alpha.-Bergamotene	209,911
26.318	Benzenemethanol, .alpha.-(1-ethenylpentyl)-.alpha.-methyl-	92,881
26.945	Arabino-Hex-1-enitol, 1,5-anhydro-2-deoxy-	102,198
27.845	Oxiranemethanol, 2-phenyl-	190,581
27.871	Cyclopropane, 1-bromo-2,2,3,3-tetramethyl-1-prop-1-ynyl-	49,869
28.261	d-Menthol	142,424.5
28.595	2-Bromopropionic acid, 2-pentyl ester	79,505
29.070	Cycloisolongifolene	120,477
30.001	2,6-Octadienal, 3,7-dimethyl-, (Z)-	535,579.5
30.495	2-Methyl-l-methylmannopyranoside	167,380
30.806	3-Cyclohexene-1-methanol, .alpha.,.alpha.,4-trimethyl-, acetate	635,343
30.824	.alpha.-Terpineol	702,887
31.110	1-Cyclohexyl-2,2-dimethyl-1-propanol acetate	79,379
31.364	L-.alpha.-Terpineol	953,500.5
31.644	Acetic acid, 1-(R)-phenylethyl ester	152,399.5
32.078	(-)-Carvone	238,609
32.618	.beta.-Bisabolene	292,323
32.907	2,6-Octadienal, 3,7-dimethyl-, (Z)-	517,386.5
33.259	Linalyl acetate	180,568.5
33.570	7,8-Dibromo-4,4,7-trimethyl-hexahydro-benzo[1,3]dioxin-2-one	31,710
33.899	Butanoic acid, 3-methyl-	116,192
34.202	Methyl salicylate	140,347
34.653	2,6-Octadien-1-ol, 2,7-dimethyl-	735,215
34.653	Benzene, 1-(1,5-dimethyl-4-hexenyl)-4-methyl-	439,300
35.221	6-Octen-1-ol, 3,7-dimethyl-, (R)-	41,711
35.225	Citronellol	91,789
35.688	Ethanol, 2-(2-butoxyethoxy)-	238,659.5
36.222	2,6-Octadien-1-ol, 2,7-dimethyl-	98,600
36.242	2,6-Octadiene, 3,7-dimethyl-1-(2-propenyloxy)-	208,993
36.563	Anethole	84,862
36.595	Estragole	89,942
37.181	p-Mentha-1(7),8-dien-2-ol	133,232
37.554	1,4-Methanobenzocyclodecene, 1,2,3,4,4a,5,8,9,12,12a-decahydro-	127,758
37.742	Butanoic acid, 3-methyl-	566,726
37.757	Heptanoic acid	847,338
37.870	2,6-Nonadienal, (E,Z)-	263,050
38.143	2,5-Pyrrolidinedione, 1-ethyl-	108,795
38.328	Benzyl alcohol	130,915.5
39.218	Phenylethyl Alcohol	42,435
39.663	Acetic acid, 6,6-dimethyl-2-methylene-7-(3-oxobutylidene)oxepan-3-ylmethyl ester	76,029
39.671	trans-.beta.-Ionone	110,928
40.302	Isoaromadendrene epoxide	90,637
40.799	11-(2-Cyclopenten-1-yl)undecanoic acid, (+)-	66,300.5
40.980	Acetic acid, 6,6-dimethyl-2-methylene-7-(3-oxobutylidene)oxepan-3-ylmethyl ester	79,824
43.179	Octanoic acid	184,543.5
46.331	Phenol, 2-ethyl-4,5-dimethyl-	50,752
46.839	2,4,6-Octatrien-1-ol, 3,7-dimethyl-(E,E)-	36,117
47.457	1,2-Cyclohexanediol, 1-methyl-4-(1-methylethenyl)-	118,730
47.870	2(4H)-Benzofuranone, 5,6,7,7a-tetrahydro-4,4,7a-trimethyl-	147,328.5
48.345	Cyclobutene, 4,4-dimethyl-1-(2,7-octadienyl)-	164,733
49.926	Benzoic acid	163,139

**Table A8:** Results for the EBB sample fingerprint determination with indication of the retention time (RT) and abundance mean for every identified compound.

RT	Compound Name	Abundance Mean
0.840	Glycidol	75,856.5
0.950	.alpha.-Chloroacrylic acid	59,320
1.530	2-Pentanol, 3-chloro-4-methyl-, (R*,R*)-(.+/−.)-	299,363
1.560	1-Methoxy-3-methyl-3-butene	289,385
3.484	.beta.-Myrcene	64,340
5.194	.beta.-Myrcene	1,191,105.5
5.938	D-Limonene	4,419,800.5
6.681	3-Hexenal, (Z)-	472,247.5
6.964	N-(Trifluoroacetyl)-N,O,O′,O″-tetrakis(trimethylsilyl)norepinephrine	122,798
7.596	Tricyclo[2.2.1.0(2,6)]heptane, 1,7,7-trimethyl-	510,285
7.607	4-Carene, (1S,3R,6R)-(-)-	531,680
8.187	1,3,7-Octatriene, 3,7-dimethyl-	520,381.5
8.550	p-Cymene	182,369
9.023	(+)-4-Carene	47,706
10.463	Dodecane, 2-methyl-	128,492
10.481	Nonane	91,987
10.740	Undecane, 3,7-dimethyl-	45,039
11.600	Sulfurous acid, hexyl pentadecyl ester	39,988
11.911	5-Hepten-2-one, 6-methyl-	99,068
11.943	1-Hepten-6-one, 2-methyl-	95,405
13.370	1-Butanol, 3-methoxy-	97,327.5
14.450	3-Hexen-1-ol	49,402
14.475	3-Hexen-1-ol, (E)-	58,769
14.669	Nonanal	112,987
15.135	Oxalic acid, allyl tetradecyl ester	53,901
15.617	Decane, 2-methyl-	41,084
15.875	2-Allyloxy-4,6-bis-phenylsulfanyl-[1,3,5]triazine	37,390
15.952	3-Decene	61,937
15.952	1-Hexanol, 3-methyl-	87,038
17.110	2-Furanmethanol, 5-ethenyltetrahydro-.alpha.,.alpha.,5-trimethyl-, cis-	46,232
17.569	Undecanol-4	52,905.5
18.168	Ammonium acetate	686,834
18.610	Furfural	171,566
18.646	Furan-2-carbohydrazide, N2-(1-methylhexylideno)-	191,841
19.639	3-Oxatricyclo[4.2.0.0(2,4)]octan-7-one	65,832
21.010	Benzaldehyde	113,463.5
21.766	Tetradecane, 4-ethyl-	42,793
22.163	1,7-Nonadiene, 4,8-dimethyl-	635,615
23.237	Citronellyl butyrate	720,225
23.251	1,2-Dihydrolinalool	778,269
23.565	Cyclohexane, 1-methyl-4-(2-hydroxyethyl)-	104,217
24.198	Linalyl acetate	20,228,501.5
24.811	trans-.alpha.-Bergamotene	91,781
25.091	Fenchol, exo-	39,940
25.813	(+)-(E)-Limonene oxide	64,200
27.380	3,4-Dimethyl-2-prop-2-enyl-2,5-dihydrothiophene 1,1-dioxide	66,881
27.385	Hotrienol	67,814
27.856	1-(Phenylmethyl)-1,2,3,6-tetrahydropyridin-3-ol	122,925
27.880	Oxiranemethanol, 2-phenyl-	180,446
28.535	10-Heptadecen-8-ynoic acid, methyl ester, (E)-	52,188
28.960	4-Pentenoic acid, 2-acetyl-, ethyl ester	785,25
30.035	2,6-Octadienal, 3,7-dimethyl-, (Z)-	367,457
30.069	Longipinene epoxide	249,326
30.305	Sulfurous acid, hexyl pentadecyl ester	200,957
30.358	Valeric acid, 3-pentadecyl ester	89,012
30.832	.alpha.-Terpineol	566,609
31.429	.alpha.-Terpineol	2,871,361
31.445	L-.alpha.-Terpineol	3,859,251
32.063	(-)-Carvone	168,274.5
32.623	.beta.-Bisabolene	183,784
32.918	2,6-Octadienal, 3,7-dimethyl-, (Z)-	153,040.5
33.281	1-Bromo-3,7-dimethyl-2,6-octadiene	539,860
33.285	Linalyl acetate	735,657
33.613	2H-Pyran-3-ol, 6-ethenyltetrahydro-2,2,6-trimethyl-	78,347
33.935	Propanedioic acid, propyl-	60,942
34.239	Methyl salicylate	44,184
34.260	Tricyclo[7.1.0.0[1,3]]decane-2-carbaldehyde	53,084
34.652	2,6-Octadien-1-ol, 2,7-dimethyl-	764,472
34.935	2H-Pyran-3-ol, 6-ethenyltetrahydro-2,2,6-trimethyl-	93,514.5
35.255	2-Pentadecyn-1-ol	70,853
35.255	9,15-Octadecadienoic acid, methyl ester	47,285
35.697	Ethanol, 2-(2-butoxyethoxy)-	110,812.5
36.251	2,6-Octadien-1-ol, 2,7-dimethyl-	618,537.5
37.164	Dihydrocarvyl acetate	78,561
37.200	2-Cyclohexen-1-ol, 2-methyl-5-(1-methylethenyl)-, acetate, (1R-cis)-	79,721
37.889	trans,cis-2,6-Nonadien-1-ol	1,039,600
37.891	2,6-Nonadienal, (E,Z)-	1,143,942
38.153	2,5-Pyrrolidinedione, 1-ethyl-	129,426.5
38.335	Benzyl alcohol	119,005
38.380	Benzene, (2,2-dimethylbutyl)-	99,835
38.721	Geranyl acetate, 2,3-epoxy-	163,962
39.235	Phenylethyl Alcohol	75,575
39.670	Acetic acid, 6,6-dimethyl-2-methylene-7-(3-oxobutylidene)oxepan-3-ylmethyl ester	134,033
40.314	Isoaromadendrene epoxide	143,108
40.585	Pyruvic acid, 3-hexenyl ester	55,982
40.793	Myrcenylacetat	109,085
40.799	8,11,14-Eicosatrienoic acid, methyl ester, (Z,Z,Z)-	96,145
41.084	3(10)-Caren-4-ol, acetoacetic acid ester	176,125
41.087	1,6-Octadien-3-ol, 3,7-dimethyl-, 2-aminobenzoate	179,443
43.193	Octanoic acid	54,969
43.780	Cholestane-3,6,7-triol, (3.beta.,5.alpha.,6.beta.,7.beta.)-	100,688
44.784	Cyclododeca-5,9-dien-1-ol, 2-methyl-, (Z,Z)-	82,400
45.205	4-Piperidin-1-yl-6-(4-tetrazol-1-yl-phenoxymethyl)-[1,3,5]triazin-2-ylamine	59,889
45.626	2-Cyclohexen-1-ol, 2-methyl-5-(1-methylethenyl)-, acetate, (1R-cis)-	111,323
46.351	Phenol, 2,3,4,6-tetramethyl-	98,885
47.882	2(4H)-Benzofuranone, 5,6,7,7a-tetrahydro-4,4,7a-trimethyl-	75,435.5

**Table A9:** Results for the SBL sample fingerprint determination with indication of the retention time (RT) and abundance mean for every identified compound.

RT	Compound Name	Abundance Mean
1.084	Acetone	331,948
1.185	2-Methyl-2-(4-nitrobenzenesulfonamido)propyl N-methylcarbamate	74,669
1.210	Cyclobutane, methylene-	110,395
1.270	Ecgonine, o-pentafluoropropionyl-, pentafluoropropyl ester	84,216
1.380	Tetrahydropyran	62,535
1.430	Diallyl carbonate	72,075
1.435	2-Propen-1-amine, N-ethyl-	93,704
1.605	Cyclobutaneoctol	50,049
3.250	2-Propenamide, N-(1-cyclohexylethyl)-	72,158
3.285	Hexanal	139,699
3.361	1,2,4-Triazole, 4-[N-(2-hydroxyethyl)-N-nitro]amino-	139,316
3.555	.beta.-Pinene	432,956
3.593	Cyclohexane, 1-methylene-4-(1-methylethenyl)-	495,699
3.855	2,6-Octadiene, 3,7-dimethyl-1-(2-propenyloxy)-	52,281
3.870	Pyridine, 2-(4-pyridylmethylenamino)-	66,726
4.450	2-Keto-3-methylene-5-methyltetrahydrothiophene	54,550
5.180	1-Pentene, 5-(2,2-dimethylcyclopropyl)-2-methyl-4-methylene-	61,122
5.180	1,6,10-Dodecatriene, 7,11-dimethyl-3-methylene-	66,379
6.024	D-Limonene	12,325,691.5
6.727	2-Hexenal	147,443
7.639	.gamma.-Terpinene	163,504.5
7.720	2-(3-Methyl-but-1-ynyl)-cyclohexene-1-carboxaldehyde	77,433
8.645	Benzene, 1-methyl-3-(1-methylethyl)-	824,049.5
9.130	trans-.beta.-Terpinyl butanoate	54,444
9.689	Octanal	70,984.5
10.410	6-Chloro-2,2,9,9-tetramethyl-3,7-decadiyn-5-ol	53,045
10.838	Dodecane, 5-methyl-	66,791
11.983	1-Hepten-6-one, 2-methyl-	171,591
12.000	5-Hepten-2-one, 6-methyl-	214,793
14.540	1,3,3-Trimethylcyclopropene	71,027
14.763	Nonanal	104,120
16.748	Cyclobutane, 1,2-bis(1-methylethenyl)-, trans-	265,149.5
17.190	2-Furanmethanol, 5-ethenyltetrahydro-.alpha.,.alpha.,5-trimethyl-, cis-	76,018
17.211	cis-5-Methyl-2-isopropyl-2-hexen-1-al	57,488
17.412	Cyclobutane, 1,2-bis(1-methylethenyl)-, trans-	303,970
18.334	Ammonium acetate	216,233
18.712	Furfural	415,801
19.127	Octane, 5-ethyl-2-methyl-	100,177
19.789	2,5-Heptadiene, (E,E)-	78,358
20.498	1,1-Dodecanediol, diacetate	57,452
20.663	1-Decene, 4-methyl-	186,582
20.683	1-Hexanol, 2-ethyl-	133,849
21.128	Benzaldehyde	193,767
21.270	Decane, 3,7-dimethyl-	126,527
21.350	Heptane, 2,3,4-trimethyl-	67,035
21.509	3,5-Octadien-2-one, (E,E)-	102,421.5
21.858	Undecane, 3,7-dimethyl-	115,470
22.150	1-Decanol, 5,9-dimethyl-	92,827
22.402	Cyclobutane, 1,2-bis(1-methylethenyl)-, trans-	81,623
22.419	Cyclohexene, 1-methyl-5-(1-methylethenyl)-	98,513
22.829	1-Isopropenyl-3-propenylcyclopentane	124,022
23.281	1,2-Dihydrolinalool	101,149
23.317	Cyclopropanemethanol, .alpha.,2-dimethyl-2-(4-methyl-3-pentenyl)-, [1.alpha.(R*),2.alpha.]-	113,237
24.144	Linalyl acetate	2,220,364
24.430	2-Decenal, (Z)-	162,906
24.448	Pentane, 1-(2,2-dibromocyclopropyl)-	114,649
25.170	Oxirane, octyl-	783,66
25.384	5-Decen-1-ol, acetate, (E)-	100,866
25.437	1-(1H-Imidazol-2-yl)-ethanone	92,763
25.862	1H-Pyrrole-2-carboxaldehyde, 1-ethyl-	56,395.5
26.174	2-Pentanol, 3-chloro-4-methyl-, (R*,S*)-(.+/−.)-	245,217.5
26.420	Butyrolactone	60,795
27.010	Sulfurous acid, octyl 2-propyl ester	85,408
27.028	Sulfurous acid, dodecyl 2-propyl ester	73,132
30.159	2,6-Octadienal, 3,7-dimethyl-, (Z)-	928,389
30.420	2,5-Dihydroxyheptane	73,287
30.480	Oxetane, 2-ethyl-3-methyl-	81,149
30.640	Thiophene, 3-methylsulfonyl-	69,756
30.909	p-Mentha-1(7),8(10)-dien-9-ol	143,103
31.487	.alpha.-Terpineol	408,707
32.192	(-)-Carvone	402,266.5
33.032	2,6-Octadienal, 3,7-dimethyl-, (Z)-	1,115,315.5
33.362	Linalyl acetate	167,765.5
33.670	Cyclopropanemethanol, 2-isopropylidene-.alpha.-methyl-	55,873
33.949	cis-p-Mentha-2,8-dien-1-ol	843,89,5
34.711	2,6-Octadien-1-ol, 2,7-dimethyl-	253,900
34.970	Cyclohexene, 3-acetoxy-4-(1-hydroxy-1-methylethyl)-1-methyl-	68,155
35.301	Citronellal	77,794.5
35.581	p-Mentha-1(7),8(10)-dien-9-ol	51,607
36.309	.beta.-Myrcene	76,695.5
36.649	1-Heptadec-1-ynyl-cyclohexanol	86,850
36.697	5-Isopropenyl-1,2-dimethylcyclohex-2-enol	50,040
37.261	trans-p-mentha-1(7),8-dien-2-ol	262,206
37.270	Carveol	221,405
37.820	Butanoic acid, 3-methyl-	378,023.5
37.943	.beta.-Myrcene	279,721
38.222	2,5-Pyrrolidinedione, 1-ethyl-	221,432.5
38.401	Benzyl alcohol	196,719
38.417	Benzene, [(2-propenyloxy)methyl]-	158,666
38.773	3-hydroxy-2-methyl-5-(prop-1-en-2-yl)cyclohexanone	108,854
39.288	Phenylethyl Alcohol	64,612
39.733	Acetic acid, 6,6-dimethyl-2-methylene-7-(3-oxobutylidene)oxepan-3-ylmethyl ester	71,831
39.739	trans-.beta.-Ionone	98,394
40.358	Cyclododeca-5,9-dien-1-ol, 2-methyl-, (Z,Z)-	58,035
40.850	1-Ethynyl-1-cyclooctanol	65,930
41.063	Acetic acid, 2,6,6-trimethyl-3-methylene-7-(3-oxobutylidene)oxepan-2-yl ester	92,499
43.248	Octanoic acid	53,587
43.765	Triacetin	72,394
47.941	2(4H)-Benzofuranone, 5,6,7,7a-tetrahydro-4,4,7a-trimethyl-	107,351

**Table A10:** Results for the TBC sample fingerprint determination with indication of the retention time (RT) and abundance mean for every identified compound.

RT	Compound Name	Abundance Mean
0.715	Acetic acid, cyano-	58,385
0.814	Acetic acid, cyano-	41,951
0.890	Disulfide, isopentyl methyl	32,293
0.891	5-Aminoisoxazole	77,786
0.967	Thiocyanic acid, 5-amino-3-methyl-4-isoxazolyl ester	59,100
0.970	3-Butynoic acid	63,100
1.083	Isobutyl nitrite	180,084
1.086	Propanal, 2-methyl-	118,133
1.305	1,4-Dioxane, 2,5-dimethyl-	52,275
1.319	4-Oxa-6-hepten-2-one, 6-bromo-3-methyl-	76,789
1.452	Oxalic acid, butyl propyl ester	205,128
1.486	Butanal, 2-methyl-	454,443
1.495	Oxirane, trimethyl-	64,342
1.520	Neburon	152,396
1.590	Carbonic acid, allyl 2-ethoxyethyl ester	85,342
1.690	Carbonic acid, allyl isohexyl ester	65,883
1.695	1,2-Pentadiene, 4,4-dimethyl-	47,310
1.750	2-Oxazolamine, 4,5-dihydro-5-(phenoxymethyl)-	50,644
1.998	Butanal, 3-methyl-	97,682
2.602	Octane, 2,3,6,7-tetramethyl-	46,275
3.302	Hexanal	153,104.5
3.375	(1RS)-propanol, 1-cyano-(2S)-(tert.butyloxycarbonyl)amino-	62,614
3.450	4-Chloro-3-methylbut-2-en-1-ol	101,437
3.485	3,4-Dimethylcyclohexanol	42,683
5.165	Cyclopropanemethanol, 2-isopropylidene-.alpha.-methyl-	42,196
5.219	Sulfide, cyclopentyl isopropyl	70,260
6.045	3-Cyclohexene-1-methanol, .alpha.,.alpha.,4-trimethyl-, acetate	33,625
6.732	2-Hexenal	213,047
6.744	3-Hexenal, (Z)-	523,559
6.760	Heptanonitrile	257,029
6.998	2,6-Nonadienal, (E,Z)-	40,726
7.370	Homopiperazine	32,993
8.270	4-Methyl-2-oxopentanenitrile	45,025
8.297	1-Pentanol	91,705
8.478	Sulfurous acid, hexyl octyl ester	64,375
8.941	Cyclohexane, 1,2,4-tris(methylene)-	30,691
10.065	1,3-Pentanedione, 2,4-dimethyl-1-phenyl-	50,566
11.997	1-Hepten-6-one, 2-methyl-	43,920
12.959	Spiro[3.5]nona-5,7-dien-1-one, 5,9,9-trimethyl-	40,729
13.104	2-Trifluoroacetoxydodecane	38,203
14.530	Cycloheptano[d]imidazolidine, 1,3-dihydroxy-2-methyl-	73,913
14.567	3-Hexen-1-ol, (E)-	106,729.5
14.605	1,3,2-Dioxaphospholane, 2-cyclohexyl-4,5-dimethyl-	56,722
14.720	1-Cyclohexylethanol	38,396
14.763	Nonanal	119,865
14.770	1,2,4-Triazol-5-acetic acid, 3-amino-	50,306
14.805	5-t-Butyl-cycloheptene	36,888
15.219	Oxirane, 2-butyl-3-methyl-, cis-	47,050
15.665	3,6-Heptanedione	166,183
15.694	Dodecane, 2-methyl-	406,601
15.706	Decane, 2,4-dimethyl-	203,345
15.790	2-Furannonanoic acid, 5-(21,23-dimethylpentacosyl)tetrahydro-, methyl ester	45,108
15.825	Piperidine-4-carboxamide, 1-(3,4,5-trimethoxybenzoyl)-	47,258
15.845	5-Bromo-1-hexene	75,733
15.885	.alpha.-Chlorocyclooctanone oxime	66,635
15.910	7-Octenoic acid, methyl ester	39,237
16.203	Benzene, 1-ethyl-3,5-dimethyl-	41,469
18.342	Ammonium acetate	102,343
18.415	Acetic acid	64,804
18.729	2-Nonenal, 8-oxo-	173,371
18.749	4-Pentenoic acid, 2-methylene-, methyl ester	182,615
18.785	6-Tetradecanol	49,218
20.671	1-Hexanol, 2-ethyl-	721,868
21.081	Benzaldehyde	163,232.5
21.305	3(2H)-Furanone, dihydro-5-isopropyl-	65,684
21.495	1,3-Butanedione, 1-(2-furanyl)-	55,641
23.285	Citronellyl butyrate	39,321
24.110	Linalyl acetate	840,872
24.413	Octanal	45,631
24.423	Cyclopropane, 1-heptyl-2-methyl-	64,716
25.172	Oxirane, dodecyl-	94,251.5
26.065	3-Isopropylidene-5-methyl-hex-4-en-2-one	33,496
26.418	Butyrolactone	47,210
27.021	Decane, 2,4,6-trimethyl-	80,426
27.023	Sulfurous acid, dodecyl 2-propyl ester	91,716
27.940	2,4,6-Cycloheptatrien-1-one, 4-methyl-	88,296
28.746	Tricyclo[3.3.1.1(3,7)]decane, 2-nitro-	276,194.5
29.028	Dispiro[4.2.4.2]tetradecane	72,071
29.038	Tricyclo[3.3.1.1(3,7)]decane, 2-nitro-	117,874
30.175	1,3-Pentadiene, 5-(2,2-dimethylcyclopropyl)-2,4-dimethyl-, (Z or E)-	60,534
30.229	.alpha.-Chlorocyclooctanone oxime	35,753
30.524	Aziridinone, 1-(1,1-dimethylethyl)-3-tricyclo[3.3.1.1(3,7)]dec-1-yl-	73,998
30.533	8,11,14-Eicosatrienoic acid, methyl ester, (Z,Z,Z)-	93,089
31.424	.alpha.-Terpineol	47,609
32.351	Dispiro[4.2.4.2]tetradecane	367,543.5
33.945	Pentanoic acid	44,424
34.295	Methyl salicylate	132,619.5
34.425	Benzenamine, N-[4-(1-methylethyl)benzylidene]-4-(1-pyrrolidylsulfonyl)-	40,434
34.685	2,6-Octadien-1-ol, 2,7-dimethyl-	93,110
34.986	Cyclopropanemethanol, 2-isopropylidene-.alpha.-methyl-	81,101
35.015	2H-Pyran-3-ol, 6-ethenyltetrahydro-2,2,6-trimethyl-	107,522
35.648	1-Phenyl-2-butanone	77,853.5
37.437	1,4-Methanobenzocyclodecene, 1,2,3,4,4a,5,8,9,12,12a-decahydro-	182,739.5
37.818	Heptanoic acid	327,611
37.826	Formic acid, 2-methylpropyl ester	270,250
38.221	2,5-Pyrrolidinedione, 1-ethyl-	111,221.5
38.402	Benzyl-diseryl phosphate	132,761
38.403	Benzyl alcohol	126,413
39.052	Pregnane-3,8,12,14,17,20-hexol, (3.beta.,5.alpha.,12.beta.,14.beta.,17.alpha.,20S)-	34,151
39.285	Phenylethyl Alcohol	55,509
39.726	trans-.beta.-Ionone	77,701
39.727	3-Buten-2-one, 4-(2,6,6-trimethyl-1-cyclohexen-1-yl)-	97,960
40.852	11-(2-Cyclopenten-1-yl)undecanoic acid, (+)-	52,398
41.019	Pentane, 2-bromo-	31,014
41.044	Acetic acid, 6,6-dimethyl-2-methylene-7-(3-oxobutylidene)oxepan-3-ylmethyl ester	78,871
43.085	3-Hepten-2-one, O-methyloxime	33,472
44.698	1,5-Hexadiene, 2,5-dipropyl-	38,397
47.938	2(4H)-Benzofuranone, 5,6,7,7a-tetrahydro-4,4,7a-trimethyl-	105,392
52.435	Methyl 4,6-benzylidene-3-deoxy-4-hexopyranoside	41,183
52.725	2-(Imidazole-1-sulfonyl)-benzoic acid methyl ester	34,590

**Table A11:** Results for the WBL sample fingerprint determination with indication of the retention time (RT) and abundance mean for every identified compound.

RT	Compound Name	Abundance Mean
0.103	Borane carbonyl	72,523
0.770	Acetic acid, cyano-	119,964
0.840	aS-Triazine-3,5(2H,4H)-dione, 6-(dimethylamino)-	109,600
1.096	Acetone	194,968
2.590	Benzeneacetic acid, 2-tetradecyl ester	142,487
3.265	Hexanal	1,020,449
3.455	Allyl trifluoroacetate	100,639
6.689	2-Hexenal	165,126.5
6.982	N-(Trifluoroacetyl)-N,O,O′,O″-tetrakis(trimethylsilyl)norepinephrine	474,946.5
8.491	Dodecane, 4-methyl-	154,490
8.511	Octane, 5-ethyl-2-methyl-	209,142
9.656	Octanal	133,742
10.964	2-Heptenal, (Z)-	132,743.5
11.957	5-Hepten-2-one, 6-methyl-	3,391,388
12.325	trans-Rose oxide	65,810
14.753	Nonanal	36,0131
15.167	trans,cis-2,6-Nonadien-1-ol	166,441.5
18.350	Ammonium acetate	1,197,691.5
18.705	Furfural	245,261
19.132	Nonane, 5-(1-methylpropyl)-	192,955
19.135	Tetradecane, 4-methyl-	137,144
20.725	Tridecane, 6-methyl-	108,712
21.129	Benzaldehyde	309,445.5
21.375	Nonane, 5-(2-methylpropyl)-	90,406
24.145	5-Hepten-1-ol, 2-ethenyl-6-methyl-	355,464,5
24.473	1-Octanol	104,288
24.493	1-Nonene	128,456
25.494	(S)-(-)-1,2,4-Butanetriol, 2-acetate	89,072
25.844	2(5H)-Furanone, 5,5-dimethyl-	107,360
26.274	(S)-(+)-1,2-Propanediol	3,130,504
26.282	R-(-)-1,2-propanediol	3,474,497
27.054	Sulfurous acid, 2-propyl undecyl ester	407,374
28.037	Acetophenone	134,811
28.823	Dodecane, 2,5-dimethyl-	90,189
30.205	2,6-Octadien-1-ol, 2,7-dimethyl-	224,600
30.445	Nonane, 4,5-dimethyl-	125,045
30.657	Verbenol	121,652
31.525	.alpha.-Terpineol	345,922
31.755	2′-Ethyl-3-[(3-phenylpropionyl)hydrazono]butyranilide	86,101
32.640	Tetradecane	365,951
32.666	Eicosane, 10-methyl-	386,657
33.025	2,6-Octadienal, 3,7-dimethyl-, (Z)-	132,753
34.015	Pentanoic acid	82,703
34.325	Oxalic acid, octadecyl propyl ester	88,447
34.377	2-methyltetracosane	115,659
34.697	2-Dodecenal, (E)-	163,451
34.697	Oxalic acid, propyl tridecyl ester	104,743
34.945	4-Methyl-2-oxopentanenitrile	101,744
35.141	Heptadecane, 2,6,10,14-tetramethyl-	101,875
35.145	Nonadecane, 2-methyl-	124,979
35.319	6-Octen-1-ol, 3,7-dimethyl-, (R)-	507,414.5
35.505	Hexadecane, 2-methyl-	83,391
35.506	Heptadecane, 3-methyl-	103,232
35.765	Ethanol, 2-(2-butoxyethoxy)-	106,602.5
36.134	Eicosane	155,798
36.343	2,6-Octadien-1-ol, 2,7-dimethyl-	242,610
36.468	Cyclohexane, eicosyl-	249,746
36.651	Eicosane, 10-methyl-	298,746.5
37.665	1,4-Methanobenzocyclodecene, 1,2,3,4,4a,5,8,9,12,12a-decahydro-	72,802.5
37.838	Hexanoic acid	886,987
37.953	1-Bromo-3,7-dimethyl-2,6-octadiene	1,140,624.5
38.241	2,5-Pyrrolidinedione, 1-ethyl-	368,554.5
38.419	Benzyl-diseryl phosphate	533,200
38.420	Benzyl alcohol	754,339
38.705	Thiazolo[3,2-a]pyridinium, 3-hydroxy-2-methyl-, acetate	92,220
38.915	Methanesulfonylacetic acid	98,645
39.303	Phenylethyl Alcohol	91,829
39.585	Octane, 2,3,3-trimethyl-	109,959
39.596	Nonadecane, 2-methyl-	159,690
40.285	Benzene, (1-butyloctyl)-	115,894
40.540	Creosol	139,225.5
40.715	Hexanoic acid, 2-ethyl-	155,344
40.754	Heptanoic acid	284,358
40.871	Ethanone, 1-(1H-pyrrol-2-yl)-	163,370.5
41.055	Pregan-20-one, 2-hydroxy-5,6-epoxy-15-methyl-	107,105
41.205	Pentafluoropropionic acid, decyl ester	85,453
41.425	m-Toluic acid, 2-ethylcyclohexyl ester	84,728
41.847	Benzaldehyde, 4-methoxy-	534,873.5
42.365	3-Isopropylidene-5-methyl-hex-4-en-2-one	103,040
42.505	(+)-3-Carene, 10-(acetylmethyl)-	66,804
43.255	Octanoic acid	3,876,198
44.623	2-Pentadecanone, 6,10,14-trimethyl-	225,638
44.755	2,6-Octadiene-1,8-diol, 2,6-dimethyl-	63,621
44.755	p-Mentha-1,8-dien-7-yl acetate	96,497
45.306	3-Ethyl-1-heptyne-3-ol	106,999.5
45.515	n-Hexadecanoic acid	282,887
45.899	.alpha.-Bulnesene	79,896
46.293	Cycloheptane, 4-methylene-1-methyl-2-(2-methyl-1-propen-1-yl)-1-vinyl-	87,168
46.294	(-)-Isolongifolol, acetate	122,326
46.424	2-(p-Tolylmethyl)-p-xylene	171,310
46.430	Ethane, 1-(o-ethylphenyl)-1-phenyl-	144,701
47.579	Pyrrole-2-carboxylic acid, 4-(1-chlorodec-1-enyl)-3,5-dimethyl-, ethyl ester	98,928
47.605	n-Decanoic acid	2,589,191
47.940	2(4H)-Benzofuranone, 5,6,7,7a-tetrahydro-4,4,7a-trimethyl-	416,391.5
50.353	Benzophenone	885,368
51.520	Vanillin	684,992
52.990	1,2-Benzenedicarboxylic acid, butyl 2-ethylhexyl ester	304,646
52.991	1,2-Benzenedicarboxylic acid, bis(2-methylpropyl) ester	390,059
